# Supplementary material for: Thin, soft, wearable system for continuous wireless monitoring of artery blood pressure
Source: Nat Commun. 2023 Aug 17;14:5009. doi: 10.1038/s41467-023-40763-3 (PMC10435523; doi:10.1038/s41467-023-40763-3)
Supplement: Supplementary file 1 — Supplementary Information [file 41467_2023_40763_MOESM1_ESM.pdf]

Supplementary information for

Thin, Soft, Wearable System for Continuous Wireless Monitoring of Artery Blood Pressure

Jian Li, Huiling Jia, Jingkun Zhou, Xingcan Huang, Long Xu, Shengxin Jia, Zhan Gao, Kuanming Yao, Dengfeng Li, Binbin Zhang, Yiming Liu, Ya Huang, Yue Hu, Guangyao Zhao, Zitong Xu, Jiyu Li, Chun Ki Yiu, Yuyu Gao, Mengge Wu, Yanli Jiao, Qiang Zhang, Xuecheng Tai, Raymond H. Chan, Yuanting Zhang, Xiaohui Ma, Xinge Yu

Correspondence to:

maxiaohui@301hospital.com.cn (X. M.)

xingeyu@cityu.edu.hk (X. Y.)

The file includes:

Supplementary Notes 1 to 6

Supplementary Figures 1 to 38

Supplementary Tables 1 to 4

Supplementary Note 1: Comparison of BP monitoring technologies

Conventionally, non-invasive blood pressure is measured with a manual cuff, in which a pressure gauge is equipped to measure the air pressure inside the cuff. By inflating the manual cuff to a pressure that is higher than BP, systolic BP and diastolic BP can be recorded with the assistance of the stethoscope. While continuous BP measurements typically associate with invasive methods. Therefore, it's extremely important to develop wearable devices for continuous BP monitoring. To date, there are mainly four categories of technologies that are developed for continuous BP monitoring (**Supplementary Table 1, Supplementary Fig. 1**): (1) Optical based technology, i.e., Photoplethysmography (PPG) measures the changes in reflected light caused by volumetric variation of blood circulation for the estimation of BP; (2) Acoustic based technology, i.e., Ultrasound wall-tracking derives the changes in artery diameter by analyzing the echo signals from anterior vessel wall and posterior vessel wall to calculate continuous BP; (3) Electrical based technology, i.e., Electrodes array measures the variation of bioimpedance generated by blood propagation to predict BP; (4) Pressure sensor based technology, i.e., High precise pressure sensor, as presented in this work, are adopted to detect dynamic pressure from artery generated by blood propagation for the estimation of BP. Among them, PPG as one of the most commonly used methods, however, suffers from insufficient penetration depth, and thus are commonly used to measure the blood flow change in peripheral blood vessels, such as those in fingertips or earlobe. In comparison, Acoustic base technology is considered as the promising candidate for continuous BP monitoring due to its high penetration depth and robust sensing capability in hemodynamic parameters. Recently, totally wearable ultrasound system has been developed for BP monitoring and tissue imaging[1]. However, for continuous BP monitoring application, ultrasound wall tracking relies on the isolated DBP and SBP to calculate the vascular resistance and stiffness related coefficient β[2,3] to calibrate the device. Therefore, personalized calibration process is required for each user, in which well trained professional must be involved, and thus increase the usage barrier. Besides, high power consumption of the electrical circuit in high frequency (over 2 MHz) signal sampling and transmission increases the system bulkiness due to the need of bulky battery for long lifetime. Electrical based technology utilities the impedance changes during blood circulations to measure pulse wave, which needs only a

set of electrodes array applied upon the arteries. Although ultrathin graphene tattoo electrodes has been developed for bioimpedance measurement[4], bulky interface between electrodes and circuit, and complicated modulation and demodulation process of the sensing signal block its application in wireless and continuous BP monitoring. In comparison, pressure sensor based technology shows advances in device stability, signal processing and transmission circuit. Real wearable pressure sensor based system has been developed[5,6] for wireless, continuous BP measurement. However, there are two mainly two drawbacks in pressure sensor based systems. The first one is BP calibration, that is converting the measured continuous pulse wave into continuous BP. Recent works[5,6] adopted a transfer coefficient to map the peak and valley value of the sensor output into SBP and DBP, respectively. However, the output voltage of the pressure sensor is highly related to individual mechanical properties, such as vascular modulus and skin modulus, and experimental settings, such as back pressure level provided by the holder, which means repeated calibration process are required for each user even two times measurement for the same user due to the contribution of back pressure variation on output voltage. The second one is the poor interfacial performance between the pressure sensor and human skin. The pressure sensor needs to be tightly mounted on the skin to effectively detect the deformation caused by blood propagation, which puts high requirement on sensor encapsulation and system integration because high integration level will reduce sensor deformation in response to arterial deformation, greatly reducing measured signal quality. Strategies have been proposed to address the poor interfacial performance issue and increase system integration level, e.g., utilizing a wrist band or watch strap to provide powerful support to the pressure sensor, which, however, will significantly reduce user comfort due to high pressure level applied on the wrist.

In this work, our wireless integrated system employs a high precise, self-powered piezoelectric sensor array to detect the pressure variation of artery caused by blood propagation, providing more stable and power saving strategy compared to PPG. Besides, compared to acoustic and electrical methods, in which bulky signal sampling and processing equipment or complicated interfacial connection are required, our device provides a totally wearable interface for users, with all the components integrated into a wristband. The backpressure generation system in our devices provide powerful supporting for the sensor array to solve the common drawbacks, such as unstable interfacial

contact and resulted poor signal quality in pressure sensor based technologies.

Additionally, from the point of BP estimation algorithms, there are several commonly used algorithms BP estimation.

(1). K-Nearest Neighbor (KNN): This algorithm calculates the distance between the input values and the k nearest sample points in the training set make predictions about the output of the input values. It can be used in nonlinear data but when it comes to large amount of data, the calculation cost is high.

(2). Support vector regression (SVR): The goal of this algorithm is to find an appropriate line to approximates the input variables and predict values. It can handle nonlinear and high-dimensional data by using kernel functions, but it might be sensitive to noise and the selected parameters needs to be tuned to optimize the model.

(3). Adaptive boosting regression (ABR): This is an ensemble learning algorithm that uses a combination of weak learners to make the prediction values. It can improve generalization, but it may be sensitive to noise and anomalous data.

(4). Multiple linear regression (MLR): This algorithm uses a linear equation to predict outcomes from independent variables. It is straightforward and easy to apply, but it may not fit nonlinear data well and it needs pre-assessment of the relationship between variables.

(5). Artificial neural network (ANN): This algorithm uses a network of multiple connected neurons to learn from input data and make predictions. It can capture nonlinearity in the features, but it risks overfitting and demands a lot of data and computation.

We applied XGBoost regression algorithm for BP estimation considering the fast computation speed and high accuracy and performance. Moreover, it does not require a large amount of data for training and can be easily optimized by fine-tuning various hyperparameters and avoiding the overfitting problems that often occur in large models such as deep neural networks.”

Supplementary Note 2: Piezo response conversion.

The electromechanical coupling behavior of the piezoelectric transducer is governed the following constitutive equations[7].

$$\sigma_{ij} = \mathrm{c}_{kl}\varepsilon_{ij} - (e_{ik})^T E_j \tag{1}$$

$$D_i = e_{ik}\varepsilon_k + k_{ij}E_j \tag{2}$$

Where σ, ε, E and D stand for the stress, strain, electrical field and electrical displacement, respectively. c, e and k stand for the elastic array, piezoelectric coupling array and dielectric array, respectively. *i, j, k,* and *l* represent the spatial direction of the parameter matrix, with *i* and *j* taking the values of 1, 2, and 3, and *k* and *l* taking the values of 1, 2, 3, 4, 5, 6. For the typical piezoelectric material PZT, the constitutive equations can be expanded into following equations.

$$\begin{bmatrix} \sigma_{11} \\ \sigma_{22} \\ \sigma_{33} \\ \sigma_{23} \\ \sigma_{31} \\ \sigma_{12} \end{bmatrix} = \begin{bmatrix} c_{11} & c_{12} & c_{13} & & & \\ c_{12} & c_{22} & c_{23} & & 0 & \\ c_{13} & c_{23} & c_{33} & & & \\ & & & c_{44} & 0 & 0 \\ & 0 & & 0 & c_{55} & 0 \\ & & & 0 & 0 & c_{66} \end{bmatrix} \begin{bmatrix} \varepsilon_{11} \\ \varepsilon_{22} \\ \varepsilon_{33} \\ 2\varepsilon_{23} \\ 2\varepsilon_{31} \\ 2\varepsilon_{12} \end{bmatrix} - \begin{bmatrix} 0 & 0 & e_{31} \\ 0 & 0 & e_{32} \\ 0 & 0 & e_{33} \\ 0 & e_{15} & 0 \\ e_{15} & 0 & 0 \\ 0 & 0 & 0 \end{bmatrix} \begin{bmatrix} E_1 \\ E_2 \\ E_3 \end{bmatrix} \tag{3}$$

From the constitutive equations, following equations can be derived:

$$\sigma_{11} = c_{11}\varepsilon_{11} + c_{12}\varepsilon_{22} + c_{13}\varepsilon_{33} - e_{31}E_3 \tag{4}$$

$$\sigma_{22} = c_{12}\varepsilon_{11} + c_{22}\varepsilon_{22} + c_{23}\varepsilon_{33} - e_{32}E_3 \tag{5}$$

$$\sigma_{33} = c_{13}\varepsilon_{11} + c_{23}\varepsilon_{22} + c_{33}\varepsilon_{33} - e_{33}E_3 \tag{6}$$

$$D_3 = e_{31}\varepsilon_{11} + e_{31}\varepsilon_{22} + e_{33}\varepsilon_{33} + k_{33}E_3 \tag{7}$$

The force generated by blood propagation can be simplified as a concentrated loading applied on the piezoelectric transducer under the simple supported constraint of PDMS substrate. Therefore, the planar strain as well as stress in <1,1> direction is equal to those in <2,2> direction ($T_{11} = T_{22}$, ε_{11}

= ε_{22}). Besides, for the PZT utilized in this work, the piezoelectric constant and elastic constant in planar direction is equal ($c_{11} = c_{22}$, $c_{13} = c_{23}$, $e_{31} = e_{32}$).

Under the simple supported constraint of the substrate, the piezoelectric response for the blood propagation is mainly generated by the flexural strain in the sensing layer. Thus, the longitudinal strain ε_{33} is assumed as zero. Consequently, **Equations (4-7)** can be simplified as follows:

$$\sigma_{11} = \sigma_{22} = c_{11}\varepsilon_{11} + c_{12}\varepsilon_{22} - e_{31}E_3 \quad (8)$$

$$\sigma_{33} = 2c_{13}\varepsilon_{11} - e_{33}E_3 \quad (9)$$

$$D_3 = 2e_{31}\varepsilon_{11} + k_{33}E_3 \quad (10)$$

For the piezoelectric transducer, the generated charge on the sandwiched electrode can be calculated with the surface integral of dielectric displacement with following equation.

$$Q = \int_A D_3 dA \quad (11)$$

Where Q and A stand for the induced charge and area of electrode, respectively.

Assuming the piezoelectric transducer maintains its original thickness, the induced voltage between the sandwiched electrode can be calculated with following equation.

$$V = \int E_3 dh \quad (12)$$

Where E3 is the electrical field along the thickness direction, h is the thickness of the PZT.

Following equations can be derived by substituting **Equation (11), (12)** into **Equation (9), (10)** and eliminating ε_{11}.

$$c_{13}\frac{Q}{A} - e_{31}\sigma_{33} = (c_{13}k_{33} + e_{33}e_{31})E_3 \quad (13)$$

Where E3 can be calculated with equation (12) as $E_3 = V / h$ and T_{33} can be calculated with the loading force F as $T_{33} = F / A$. Thus, the generated charge on the electrode can be calculated with

following equation.

$$Q = \frac{A(c_{13}k_{33} + e_{33}e_{31})}{c_{13}h}V + \frac{e_{31}}{c_{13}}F \quad (14)$$

Then, the current I in the transducer, with an external resistance R, can calculated as derivation of the induced charge on the electrode.

$$I = \frac{dQ}{dt} = \frac{A(c_{13}k_{33} + e_{33}e_{31})}{c_{13}h}\frac{dV}{dt} + \frac{e_{31}}{c_{13}}\frac{dF}{dt} \quad (15)$$

$$V = IR = R\frac{A(c_{13}k_{33} + e_{33}e_{31})}{c_{13}h}\frac{dV}{dt} + R\frac{e_{31}}{c_{13}}\frac{dF}{dt} \quad (16)$$

From **Equation (16)**, the relation between the output voltage and input loading of the piezoelectric transducer is presented. In the frequency domain, the loading force and output voltage at each frequency can be expressed as following equations [8], where ω is the angular frequency of the system.

$$F(f) = F_0(f)e^{j\omega t} \quad (17)$$

$$V(f) = V_0(f)e^{j\omega t} \quad (18)$$

By substituting **Equation (17), (18)** into **Equation (16)**, following equation can be derived.

$$V_0(f) = R\frac{A(c_{13}k_{33} + e_{33}e_{31})}{c_{13}h}V_0(f)j\omega + R\frac{e_{31}}{c_{13}}F_0(f)j\omega \quad (19)$$

Considering the loading force as the system input and the voltage as the system output, the relation between output and input of the piezoelectric system can be expressed as **Equation (20)**.

$$V_0(f) = \frac{Rj\omega\frac{e_{31}}{c_{13}}}{1 - \frac{A(c_{13}k_{33} + e_{33}e_{31})}{c_{13}h}Rj\omega}F_0(f) \quad (20)$$

The transfer function of the system can be derived from **Equation (20)**.

$$G(f)=\frac{V_0(f)}{F_0(f)}=\frac{Rj\omega\frac{e_{31}}{c_{13}}}{1-\frac{A(c_{13}k_{33}+e_{33}e_{31})}{c_{13}h}Rj\omega} \tag{21}$$

To convert the output voltage signal to loading force generated by blood propagation, so that the features of waveform of blood pressure can be well extracted, the inverse Laplace conversion is applied to **Equation (20)**. The relation between loading force and output voltage in time domain is expressed as follows.

$$F(t)=\frac{1}{R\frac{e_{31}}{c_{13}}}\int_0^t V(\tau)d\tau-\frac{A(c_{13}k_{33}+e_{33}e_{31})}{he_{31}}V(t)+C \tag{22}$$

Where C is the loading force at t = 0.

From **Equation (22)**, it is obvious that the loading force is composed of two parts, the integration of output voltage in the time range of 0-t, and the output voltage. Besides, the contribution of each part to the force is depended on the structural parameters and electromechanical coupling parameters of the piezoelectric transducer. It has been reported that the thickness is the main parameters that determines the relation between loading force and the output voltage[6]. When the thickness of PZT is around tens of microns, the output voltage plays an important role to the force, while negligible contribution when the thickness comes to hundred-micron level. Therefore, in this work, with the thickness of PZT equal to 200μm, the force in mainly contributed by the integration part of output voltage. Thus, we applied the integration of output (**Equation (23)**) to reproduce the features of BP waveform.

$$F(t)=\int_0^t V(\tau)d\tau \tag{23}$$

It is worth noting that the coefficient of the integration part contributes only to the amplitude of the loading force instead of the waveform features, and we performed amplitude normalization in the training model. Thus, the coefficient of the integration part is neglected for reproducing the features of the BP waveform.

Supplementary Note 3: Piezoelectric simulation

The finite element analysis (FEM) simulation was conducted with commercial software ABAQUA (2018) to study the electrical behaviors and mechanical deformation under a set of dynamic loading forces. The PZT was modeled with piezoelectric materials governed by the following constitutive model[7].

$$\begin{bmatrix}\sigma_{11}\\ \sigma_{22}\\ \sigma_{33}\\ \sigma_{23}\\ \sigma_{31}\\ \sigma_{12}\end{bmatrix}=\begin{bmatrix}c_{11} & c_{12} & c_{13} & & & \\ c_{12} & c_{22} & c_{23} & & 0 & \\ c_{13} & c_{23} & c_{33} & & & \\ & & & c_{44} & 0 & 0\\ & 0 & & 0 & c_{55} & 0\\ & & & 0 & 0 & c_{66}\end{bmatrix}\begin{bmatrix}\varepsilon_{11}\\ \varepsilon_{22}\\ \varepsilon_{33}\\ 2\varepsilon_{23}\\ 2\varepsilon_{31}\\ 2\varepsilon_{12}\end{bmatrix}-\begin{bmatrix}0 & 0 & e_{31}\\ 0 & 0 & e_{32}\\ 0 & 0 & e_{33}\\ 0 & e_{15} & 0\\ e_{15} & 0 & 0\\ 0 & 0 & 0\end{bmatrix}\begin{bmatrix}E_1\\ E_2\\ E_3\end{bmatrix} \tag{24}$$

$$\begin{bmatrix}D_1\\ D_2\\ D_3\end{bmatrix}=\begin{bmatrix}0 & 0 & 0 & 0 & e_{15} & 0\\ 0 & 0 & 0 & e_{15} & 0 & 0\\ e_{31} & e_{31} & e_{33} & 0 & 0 & 0\end{bmatrix}\begin{bmatrix}\varepsilon_{11}\\ \varepsilon_{22}\\ \varepsilon_{33}\\ 2\varepsilon_{23}\\ 2\varepsilon_{31}\\ 2\varepsilon_{12}\end{bmatrix}+\begin{bmatrix}k_{11} & 0 & 0\\ 0 & k_{11} & 0\\ 0 & 0 & k_{33}\end{bmatrix}\begin{bmatrix}E_1\\ E_2\\ E_3\end{bmatrix} \tag{25}$$

where σ_{ij}, ε_{ij}, E_i, and D_i stand for the stress, strain, electric field and electrical displacement in the piezoelectric material, respectively. c_{ij}, e_{ij} and k_{ij} are the electric array, piezoelectric array and dielectric array, respectively. The elastic parameters $c_{11} = c_{22}$ = 126 GPa, c_{12} = 79.5 GPa, $c_{13} = c_{23}$ = 8401 GPa, c_{33} = 117 GPa. The piezoelectric parameters e_{31} = -6.5 N/(V·m), e_{33} = 23.3 N/(V·m), e_{15} = 17 N/(V·m). The dielectric parameters k11 = 1700ε_0, k33 = 1471ε_0, where are the vacuum permittivity ($\varepsilon_0 \approx 8.85\times10^{-12}$ F/m). The silicone substrate (PDMS) was modeled by hexahedron elements (C3DR8) with the elastic modulus (*E*) and Poisson's (*v*) rate at 145 KPa and 0.49, respectively. The PI layer (25 μm), Au layer (200 nm), silver paste (20 μm) were modeled by shell elements (S4R). The mechanical parameters of PI, Au and silver paste were set as E_{PI} = 2.5 GPa, v_{PI} = 0.34, E_{Au} = 78 GPa, v_{PI} = 0.44, E_{paste} = 2.8 GPa, v_{paste} = 0.28, respectively.

Supplementary Note 4: Peak detection algorithm and signal quality assessment

After we acquire the noise-reduced signal that lasts for 5 seconds, we down sample the signal to 1000 points per second. First, we calculate the first-order differential of the sequence and assign the positive values, zero-equal and negative values of the result to -1, 0, and 1, respectively. Second, we calculate the first-order differential of the previous result and then record the indexes of the negative values. We compute the mean value ‘avg' and standard deviation ‘sd' of all points. To search for peaks from the recorded array, we set the time distance threshold to 700 between adjacent peaks based on the empirical value. Afterward, we traverse the peak indexes and retain the candidates $\mathrm{y(i)}$ that satisfy the condition $\mathrm{y(i)} - \mathrm{avg} > 1.5 \times \mathrm{sd}$; The subsequent processing consists of filtering out the peaks that are too close to each other (less than the distance), and the remaining points are the desired wave peaks. For signal quality assessment, we set the peak interval threshold Tp, their standard deviation threshold SDp and the signal kurtosis threshold T_k according to empirical values, and compare the actual values with the thresholds to determine whether the signal is available. We consider the signal as available if the following criteria are met: the relative difference between the mean peak interval and Tp is less than 25%, and both the standard deviation and the kurtosis of the signal are below their respective thresholds.

Supplementary Note 5: BP estimation performance evaluation

ME,SD,and RMSE are used to evaluate the accuracy of BP prediction performance. Pearson correlation coefficient (r) is to evaluate the linear correlation relationship between prediction values and reference values. The 95% confidence interval represents the values range covered the true values.

1. Mean error (ME):

$$\mathrm{ME} = \frac{1}{n}\sum_{\mathrm{i=1}}^{\mathrm{n}}(\mathrm{y_i} - \mathrm{x_i}) \tag{26}$$

2. Standard deviation (SD):

$$\mathrm{SD} = \sqrt{\frac{1}{\mathrm{n-1}}\sum_{\mathrm{i=1}}^{\mathrm{n}}(y_i - x_i - \mathrm{ME})^2} \tag{27}$$

3. Root mean square error (RMSE):

$$\mathrm{RMSE} = \sqrt{\frac{1}{\mathrm{n-1}}\sum_{\mathrm{i=1}}^{\mathrm{n}}(y_i - x_i)^2} \tag{28}$$

Where y_i is the prediction value, x_i is the reference value, and n is the number of samples.

4. 95% Confidence Interval (CI):

$$95\%\mathrm{CI} = [\mathrm{ME} - 1.96 \times \mathrm{SD}, \mathrm{ME} + 1.96 \times \mathrm{SD}] \tag{29}$$

Supplementary Note 6: HGCP process

Creating a wide range of blood pressure fluctuations plays a crucial role in improving the model prediction performance since the data model is not targeted on a specific BP range or individuals. Therefore, a large dynamic range is necessary to cover as many users as possible, individuals with normal BP, hypertension patients and hypotensive patients, during the model training process. Several strategies, including Valsalva maneuver and treadmill cycling, have been adopted to create a reasonable dynamic BP range[4,9]. However, they suffer from either less valuable BP range or lower number of reasonable BP data points. HGCP maneuver, in contrast, provides not only a board dynamic BP range, but also a smooth BP variation process, both of which endow the excellent prediction accuracy. Most importantly, HGCP maneuver will not cause a board fluctuation in heart rate (HR)[10], contributing to a better prediction accuracy. Specifically, the wireless wristband together with the commercial continuous BP monitoring equipment (Bio-PAC) were put on the left hand of the individuals. Then the individuals were asked to perform hand grip for 2 min to slowly elevate their BP owing to increased oxygen level required. After that, the individuals were asked to immerse their hand into ice-cold water (4 °C) for 1 min, during which the cold stimulus activates afferent sensory pathways that, in turn, trigger a sympathetic response to further increase their BP[11]. Finally, a resting period for 5 min was performed to drop their BP to baseline.

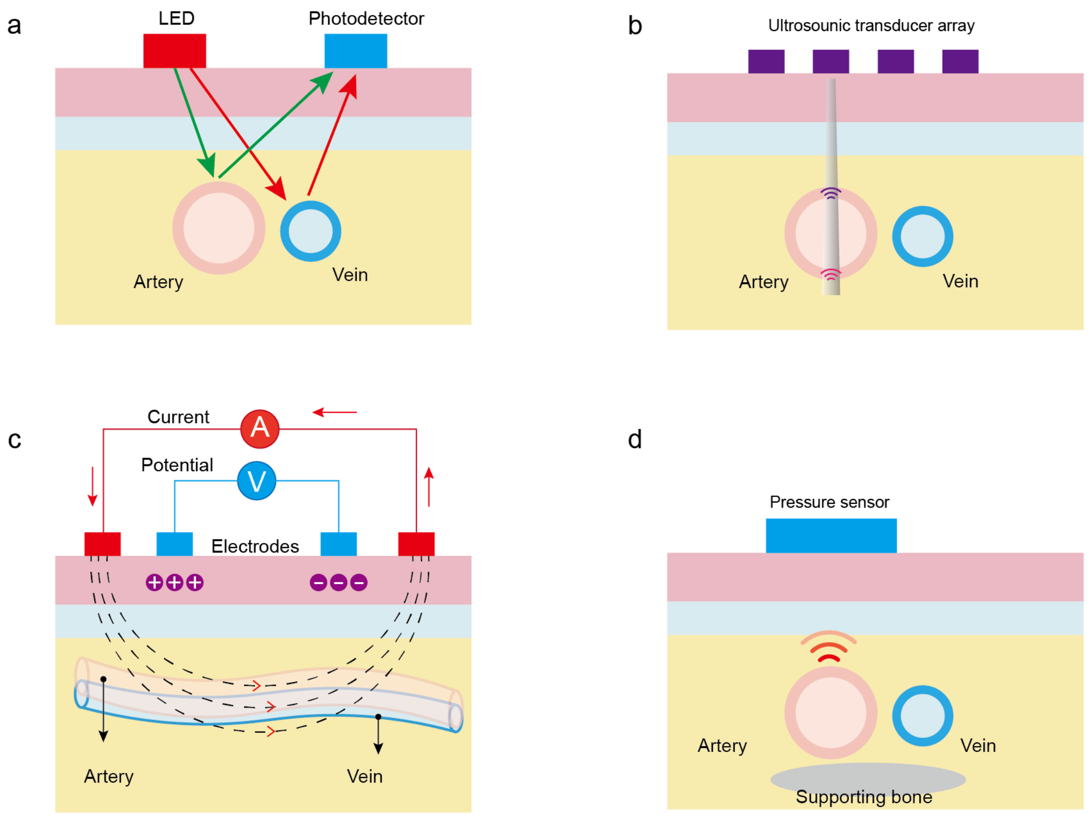

Supplementary Fig. 1. Technical comparison of different BP measurement technologies. The comparison focuses on the four types of most commonly utilized technologies, including PPG (a), ultrasound wall tracking (b), bio impedance (c) and pressure sensor (d).

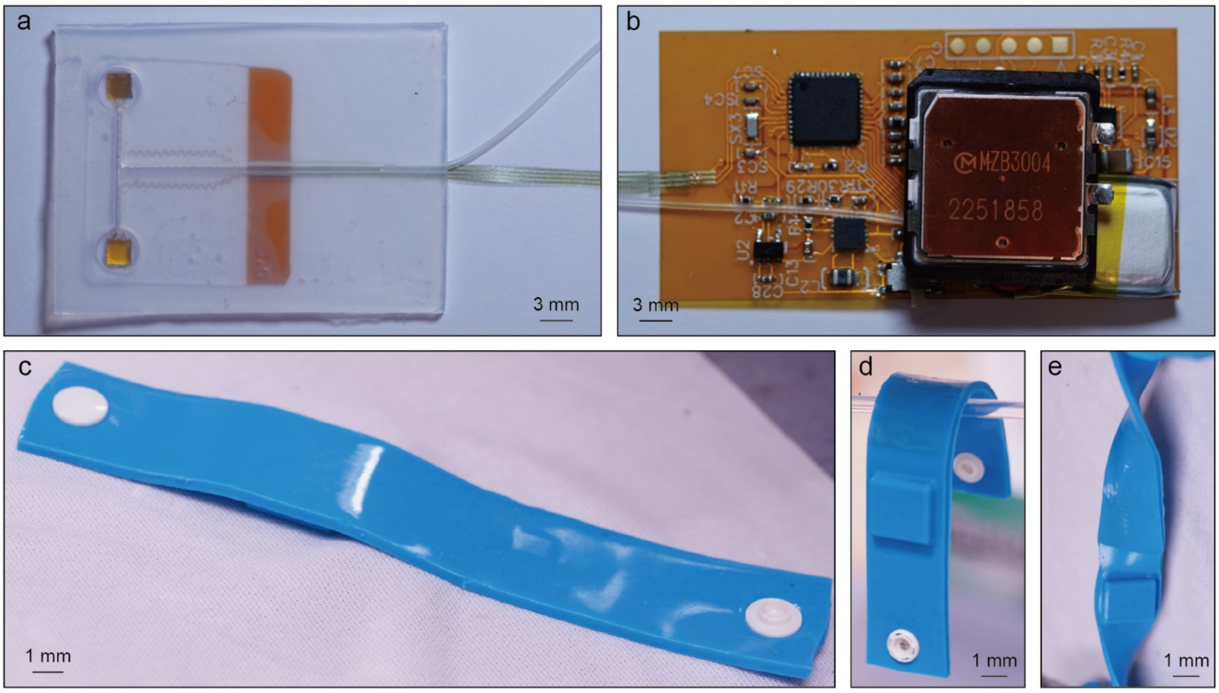

Supplementary Fig. 2. Optical images of the wireless system. (a). Optical image showing the spatial position of the pressure sensor and the micro airbag. (b). Optical image of the signal processing/transmission module and the micro pump. (c). Optical image wireless system sealed in a wireless wristband. The flexible system components endow the wireless wrist with excellent flexibility and robustness that maintains its functionality after bending deformation (d) and severe twisting deformation (e).

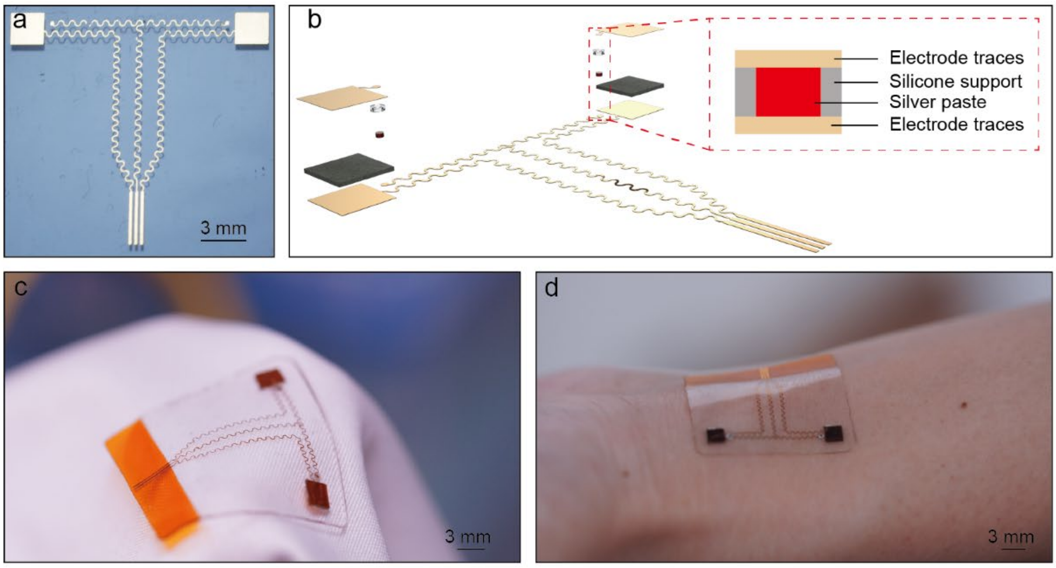

Supplementary Fig. 3. Optical images and schematics of the flexible sensor. (a). Optical image showing the bottom electrodes traces on PDMS substrate. (b). Schematic illustration of the sensor structural and integration strategy. (c). Optical images of the sensor showing excellent flexibility that can be conformally mounted on human skin (d).

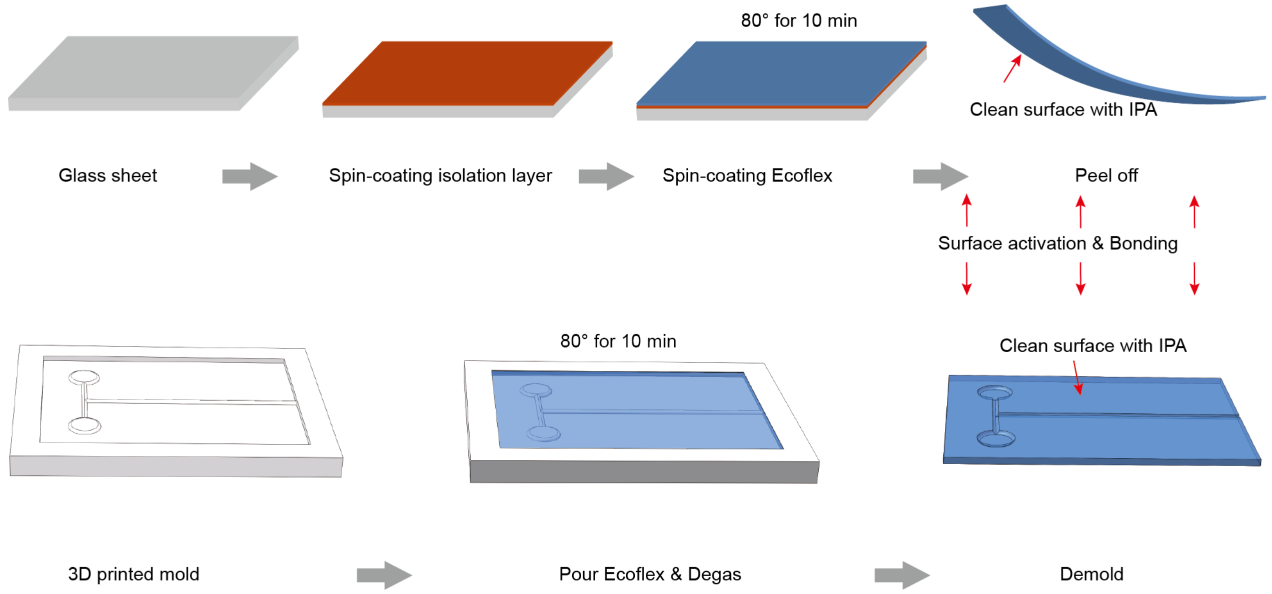

Supplementary Fig. 4. Fabrication process of the micro airbag.

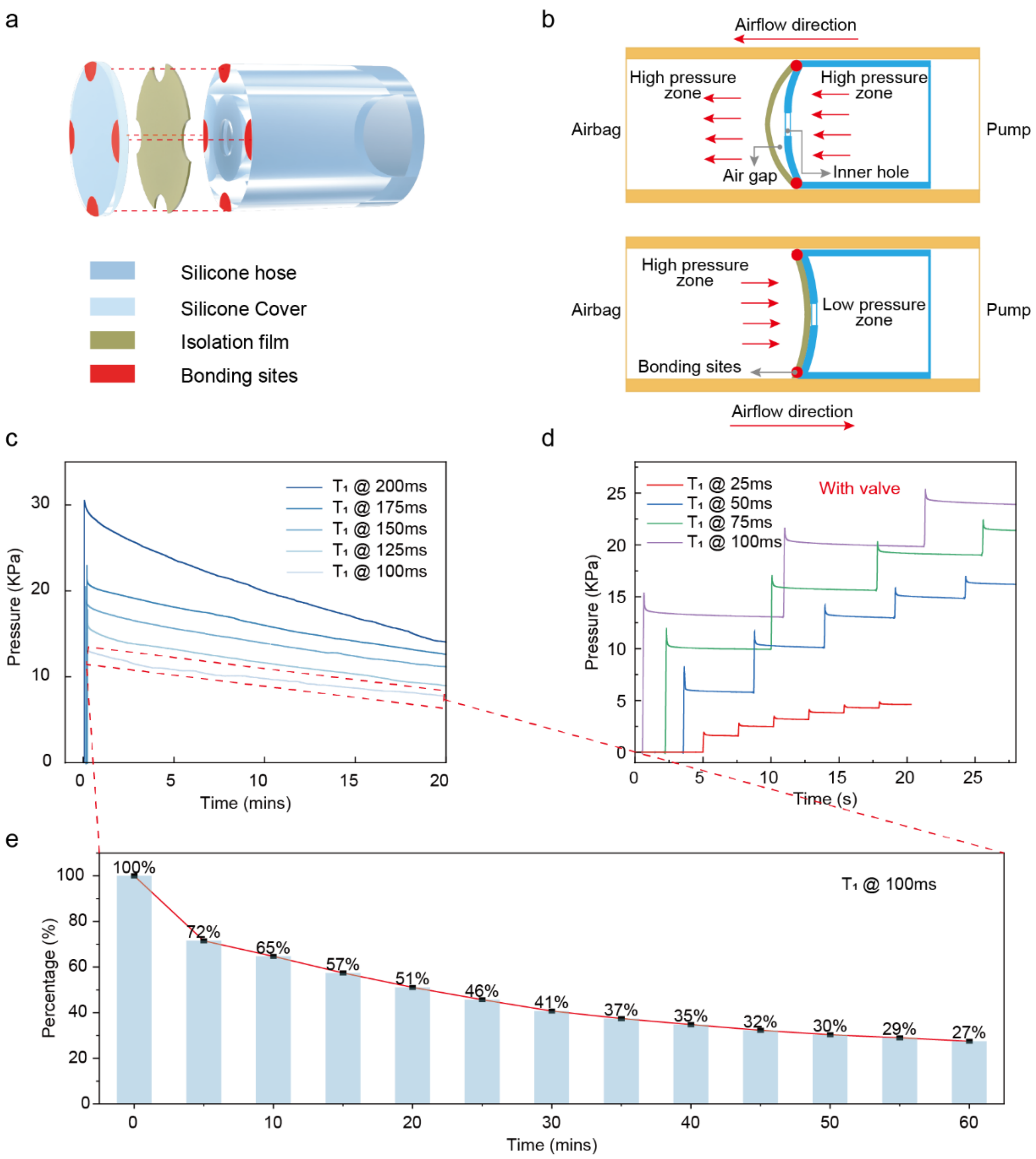

Supplementary Fig. 5. Structural design and performance characterization of the one-way valve. (a). Schematic illustration of the components and their connection of the soft one-way valve. (b). Schematic illustration of the working principle of the soft valve under inverse airflow direction. (c). Pressure in the airbag versus time after the one-way valve is equipped, with a set of pumping duration ranging from 100ms to 200ms, corresponding maximum pressure at …., respectively. (d). Pressure in the airbag versus time under multi pumping phases, with pumping time ranging from 25ms to 100ms. (e). Pressure percentage in the airbag after the pump is turn off for 60 minutes, with pumping time and maximum pressure at 100ms and 13KPa, respectively.

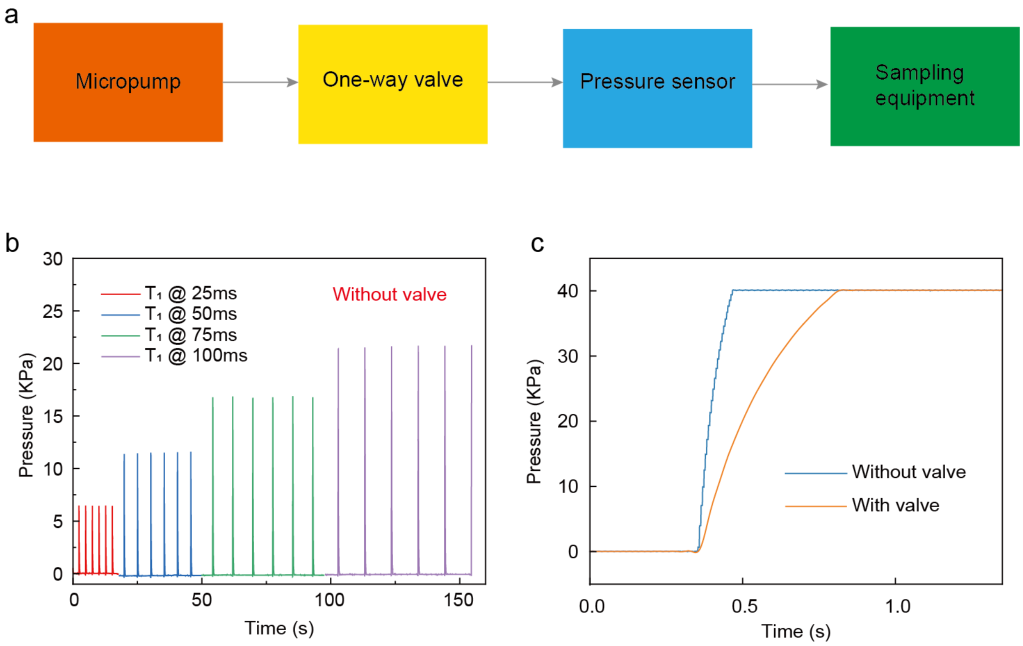

Supplementary Fig. 6. Working mode of the micro pump. (a). Schematic illustration of the system settings in pressure measurement in the pump outlet. (b). The maximum pressure of the pump under a series of pumping time, ranging from 20 ms to 100 ms. (c). Comparison of response time of the system with and without the one-way valve assembled.

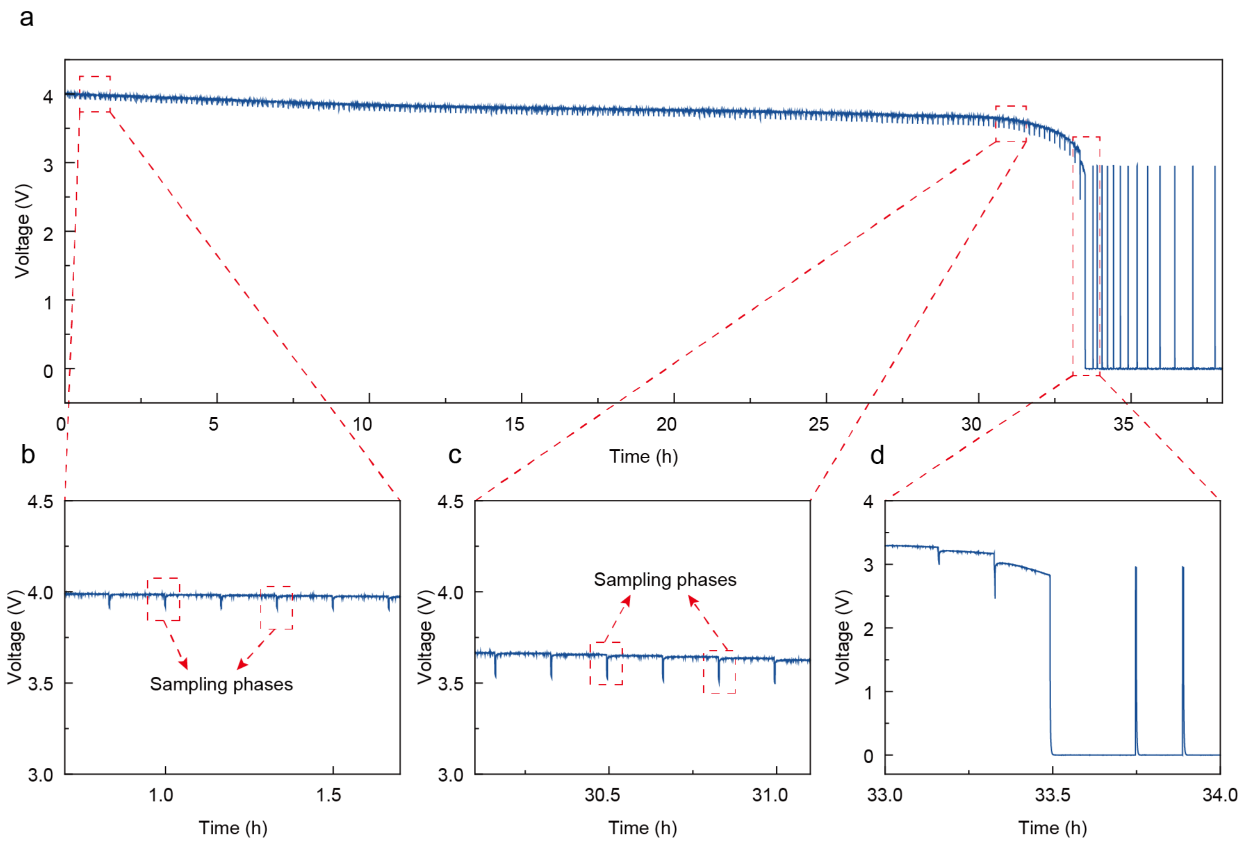

Supplementary Fig. 7. Lifetime of the rechargeable lithium-ion battery in intermittent working mode. (a). Voltage variation data showing the working duration of almost two days. (b) and (c). Voltage variation data presenting working phases at the beginning and the ending of the test, respectively. (d). Voltage data showing the system is powered down after 33.5 h sampling.

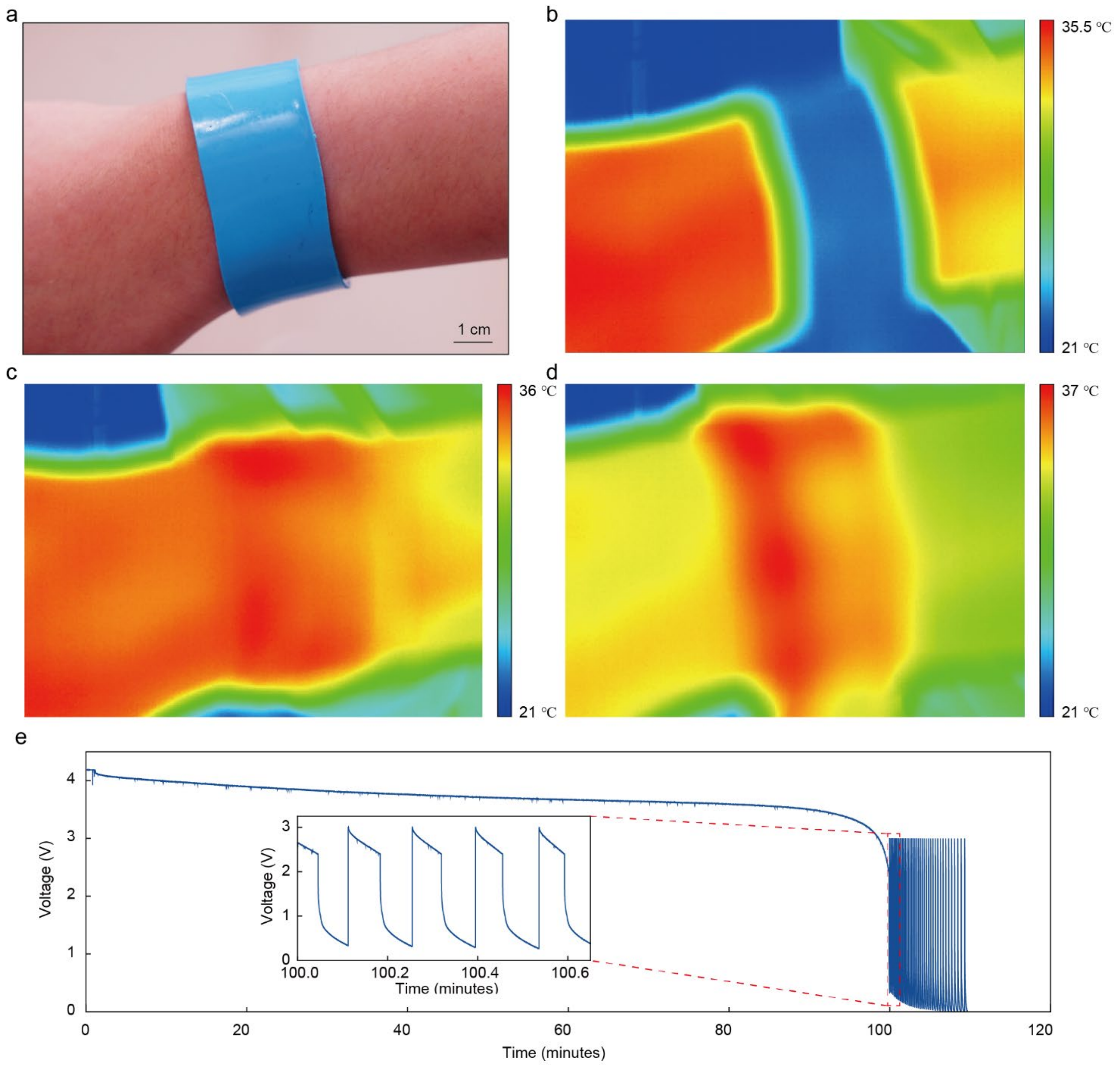

Supplementary Fig. 8. Temperature and working duration of the wireless system. (a). Optical image of the silicone wristband worn on user's wrist. (b-d). Thermal images of the silicone wristband worn on user's wrist after a set of working time, 0.5h (b), 1h (c) and 2h (d), respectively. (e). Voltage of the coin-size lithium-ion battery during working. A coin-size lithium-ion battery, with the capacity of 80mAh, can continuously power the system for 2h, with voltage of the battery changing from 4.2V to 2.8V before stopping working.

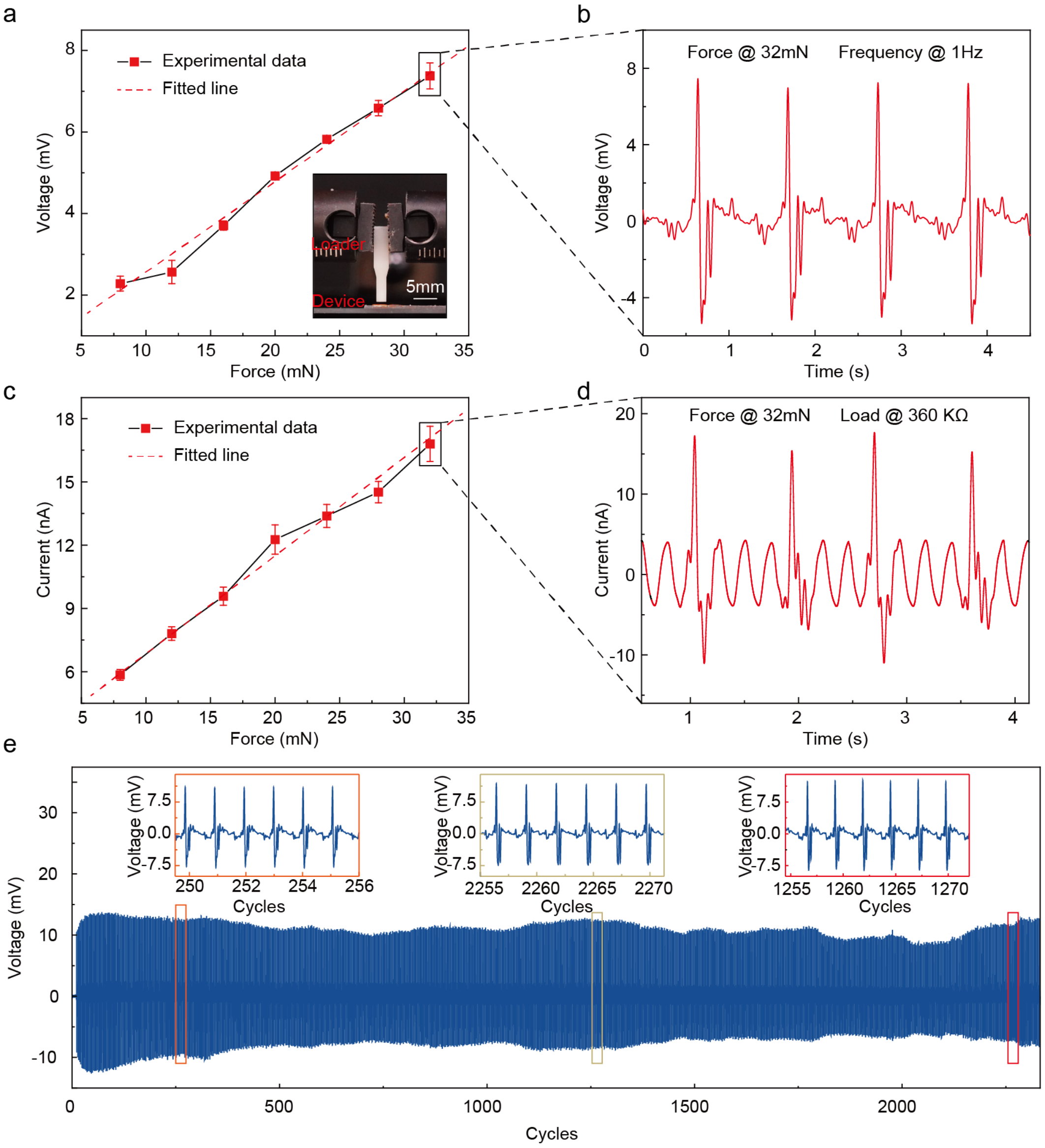

Supplementary Fig. 9. Electric characterization of the piezoelectric sensor. (a). Output voltage versus loading force of the piezoelectric sensor in the force range of 5-35 mN. The sensor presents excellent linearity with force sensitivity at 0.2 V/N. (b). Corresponding output voltage with the loading force of 32 mN at 1Hz. (c). Output current of versus loading force of the piezoelectric sensor in series with an external resistance, with resistance value at 360 KΩ. (d). Corresponding current with the loading force of 32 mN at 1Hz. (e). Output voltage of the piezoelectric sensor under the loading force of 50 mN over 2500 loading cycles. Inset figures show the excellent stability of the sensor, with stable output voltage generated after over 2500 loading cycles. n = 4 voltage peaks; center, mean; error bars, S.D. for plots of a and c.

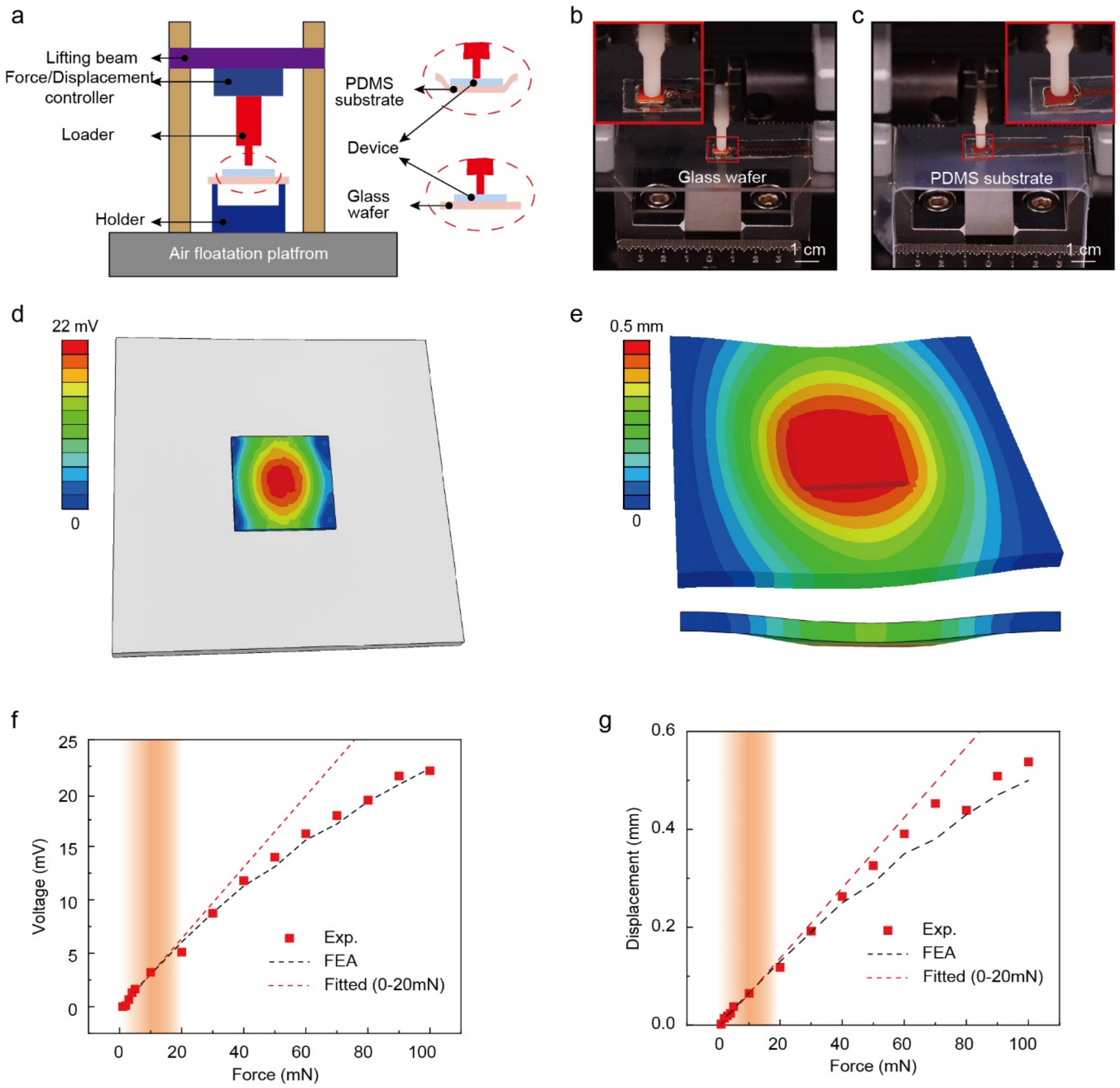

Supplementary Fig. 10. Theoretical and experiments characterization of the sensor output on soft and rigid substrate. (a). Schematic illustration of the Micro Newton Tester and device deformation on soft substrate and rigid substrate. (b) and (c). Optical images presenting the device with glass substrate (b) and PDMS substrate (c) loaded by the tester. (d). FEA result presenting the electric potential on the top surface of the piezoelectric transducer under the loading force of 100 mN. (e). FEA result showing the spatial displacement of the sensor and substrate under the loading force of 100 mN. (f) and (g). Results comparison between theoretical simulation and experiments of the surface electric potential (f) and spatial displacement (g) under a range of loading force ranging from 0 to 100 mN, where the voltage outputs show excellent consistency under the loading of pulse propagation, ranging from 0 to 20 mN.

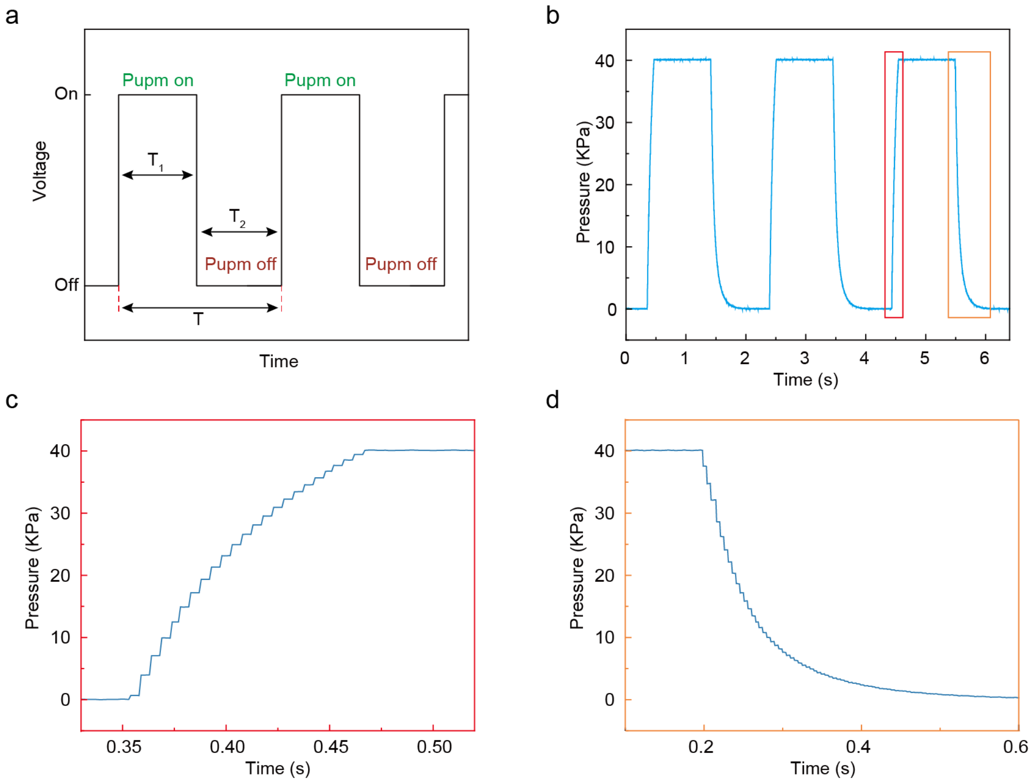

Supplementary Fig. 11. Performance characterization of the micropump. (a). Powering mode to the pump. A PWM control signal with period T (high level for T_1 and low level for T_2) was applied to the pump. (b). Pressure versus time of the pump driven by T_1 and T_2 at 1000ms. (c). Response time of the pump from zero pressure to a maximum pressure at 40KPa. (d). Response time of the pump from a maximum pressure at 40KPa to zero pressure.

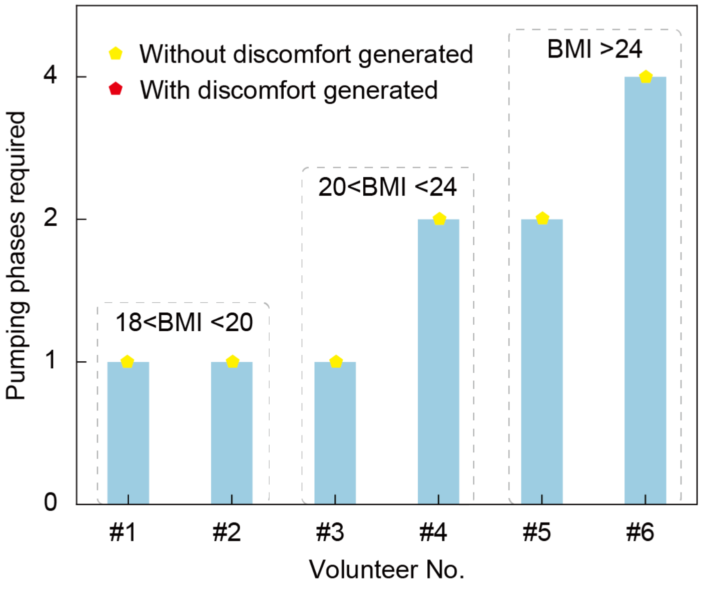

Supplementary Fig. 12. Minimum pumping phases required for effective detecting of continuous pulse wave and if discomfort feeling generated during 30 minutes continuous measurement.

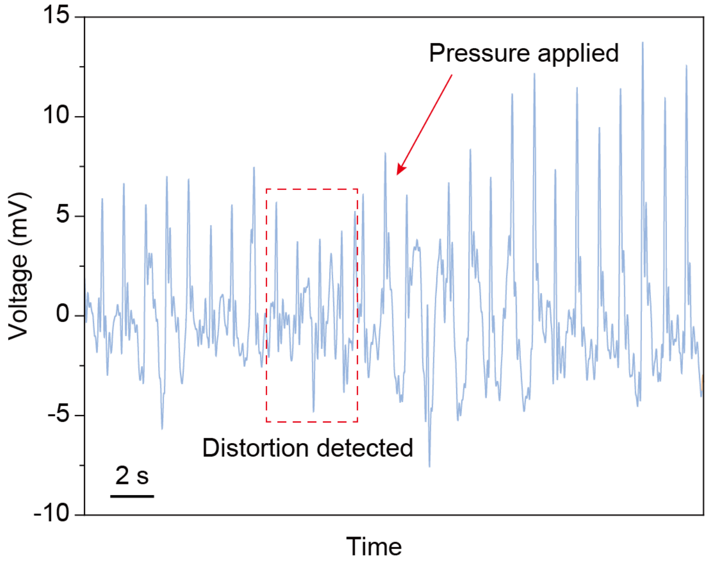

Supplementary Fig. 13. Robustness evaluation of the close-looped control strategy against signal distortion.

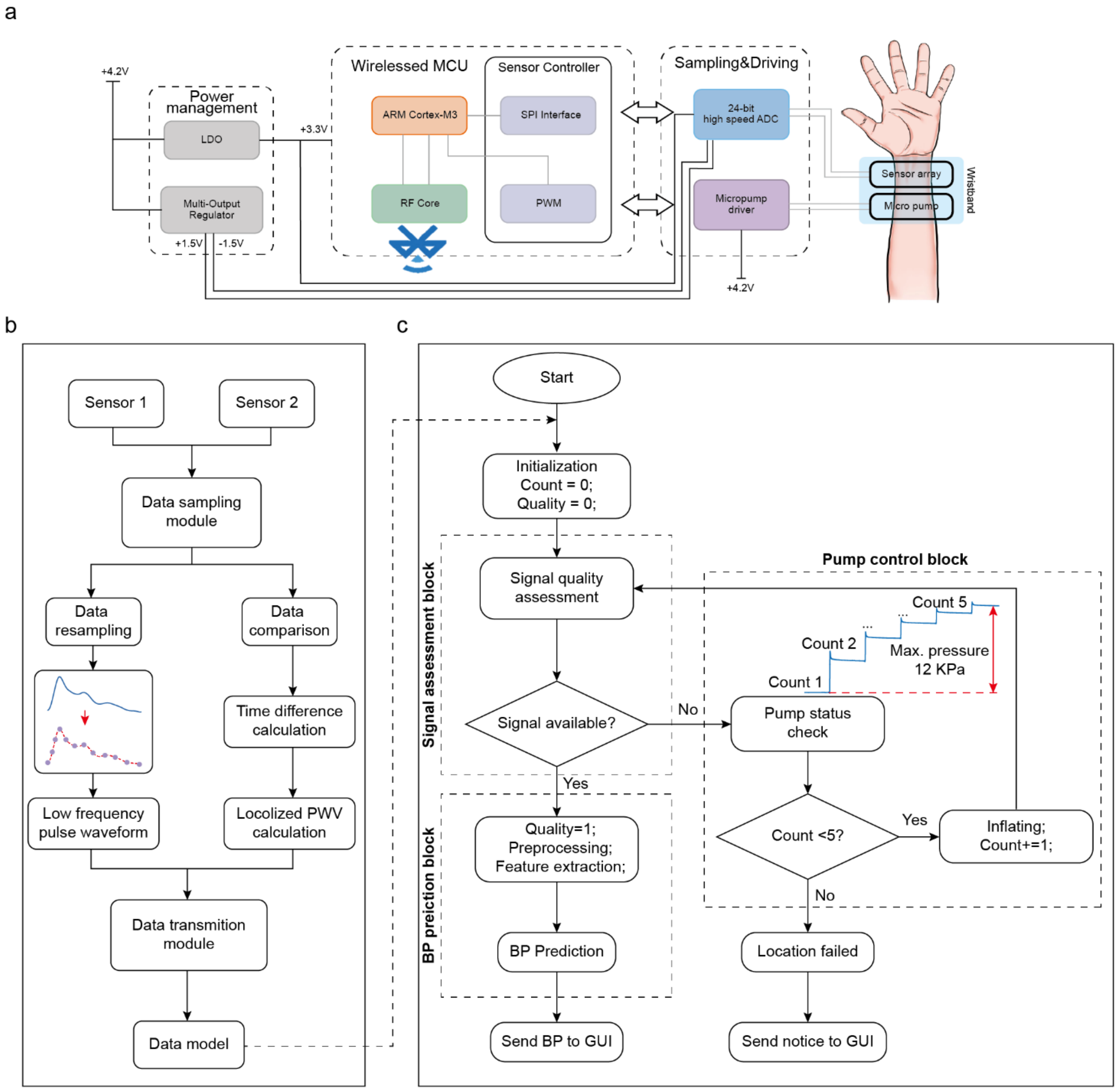

Supplementary Fig. 14. Schematic diagrams showing the system control, data processing and data transmitting. (a). Brief block diagram of the flexible circuit. (b). Block diagrams presenting the data sampling and transmission strategy. (c). Flow chart showing close-looped control strategy for the backpressure generation system, where the pumping time for each pumping phase is set as 35ms to realize a maximum pressure of 12KPa after 5 pumping phases.

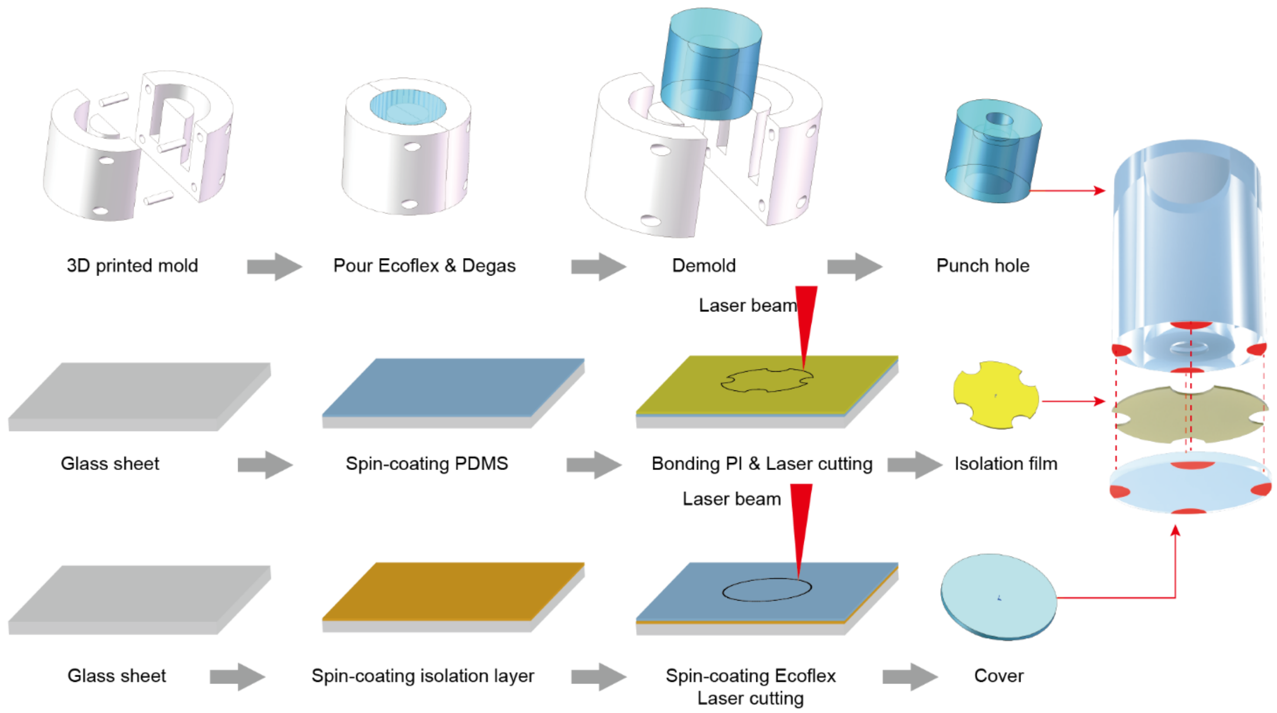

Supplementary Fig. 15. Fabrication process of the one-way valve, where three components, silicone hose, isolation film and silicone cover are fabricated separately, and then assembled with silicone glue at the pre-designed bonding sites.

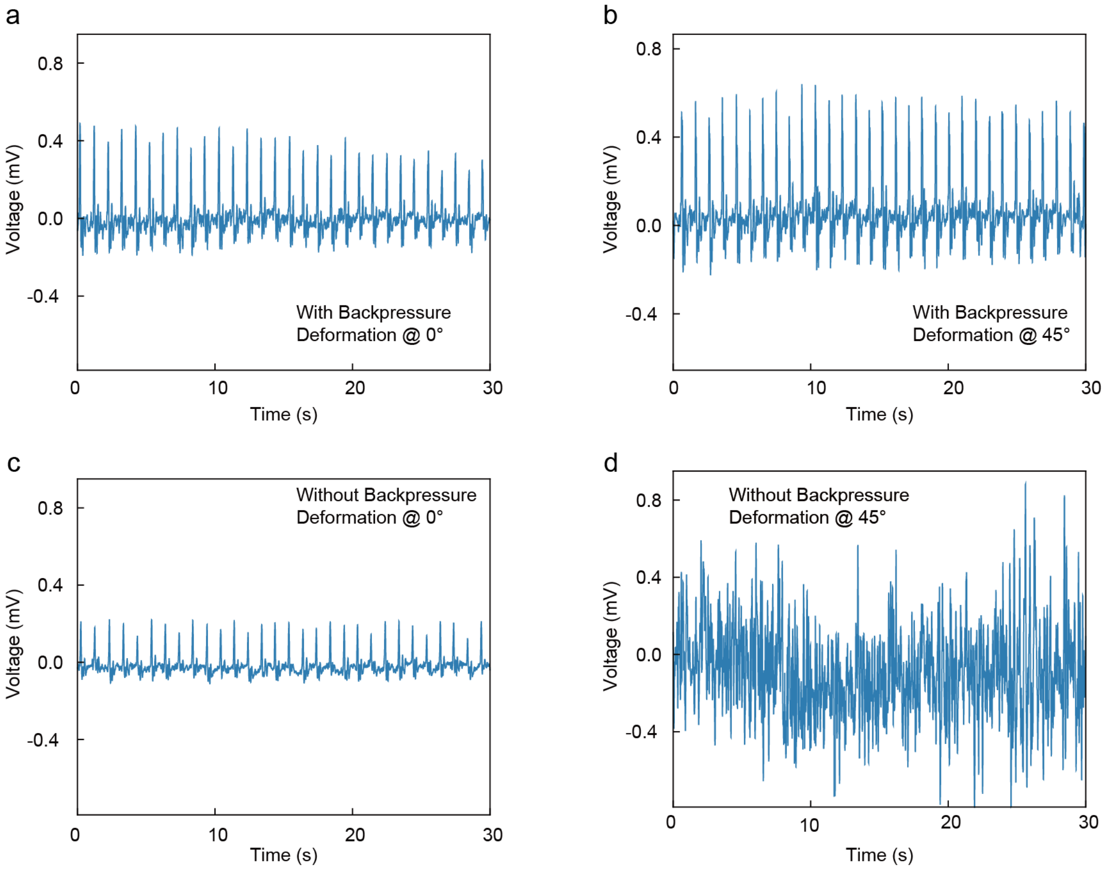

Supplementary Fig. 16. Performance comparison of the sensing module with and without the active pressure adaption unit. (a) and (b). Signals comparison of the sensor unit under 0° and 45° deformation with backpressure applied, where satisfying signals were measured regardless of deformation. (c) and (d). Signals comparison of the sensor unit under 0° and 45° deformation without backpressure applied, where although regular and stable piezo response was generated without deformation, there was no piezo response detected after the deformation (d) due to the unstable interface.

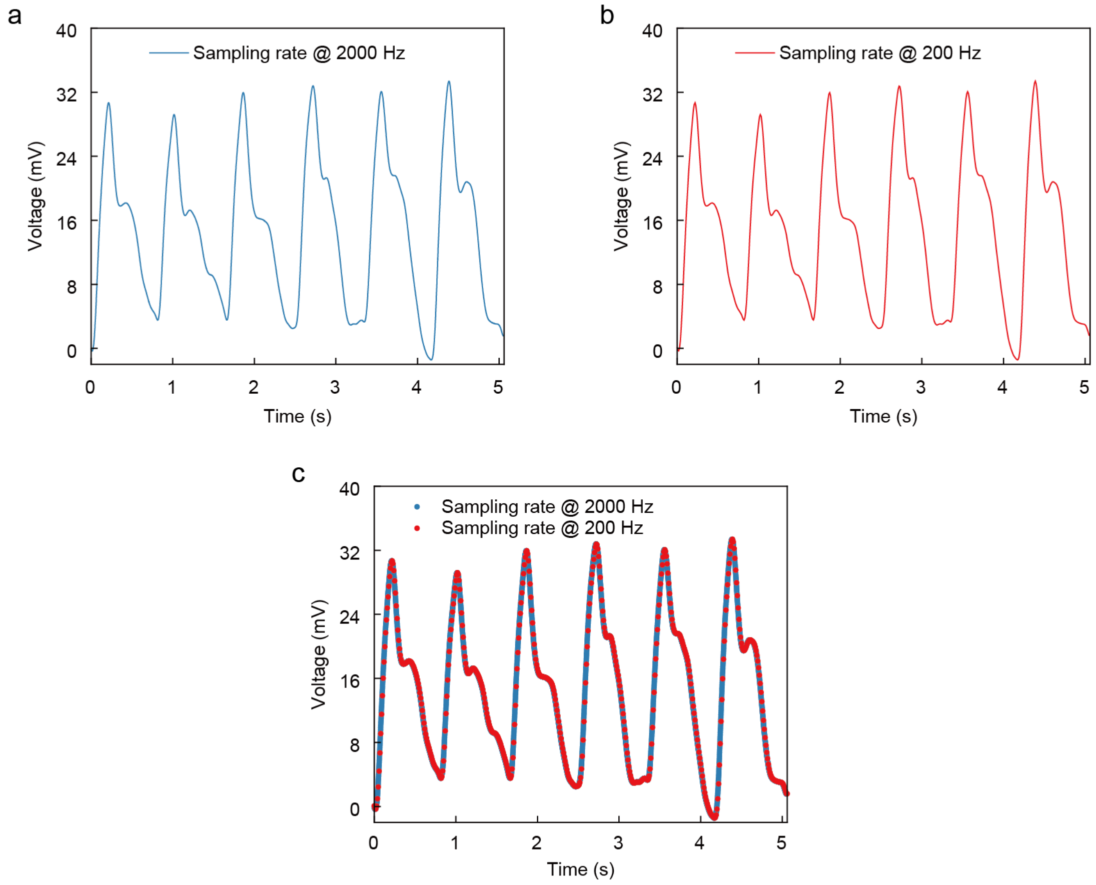

Supplementary Fig. 17. Signals comparison of the pulse wave under high and low sampling frequency. (a) and (b). Measured piezo response under high frequency (2000 Hz) and low frequency (200 Hz). (c). Waveforms comparison between signals sampled with high sampling frequency and resampled with 200 Hz.

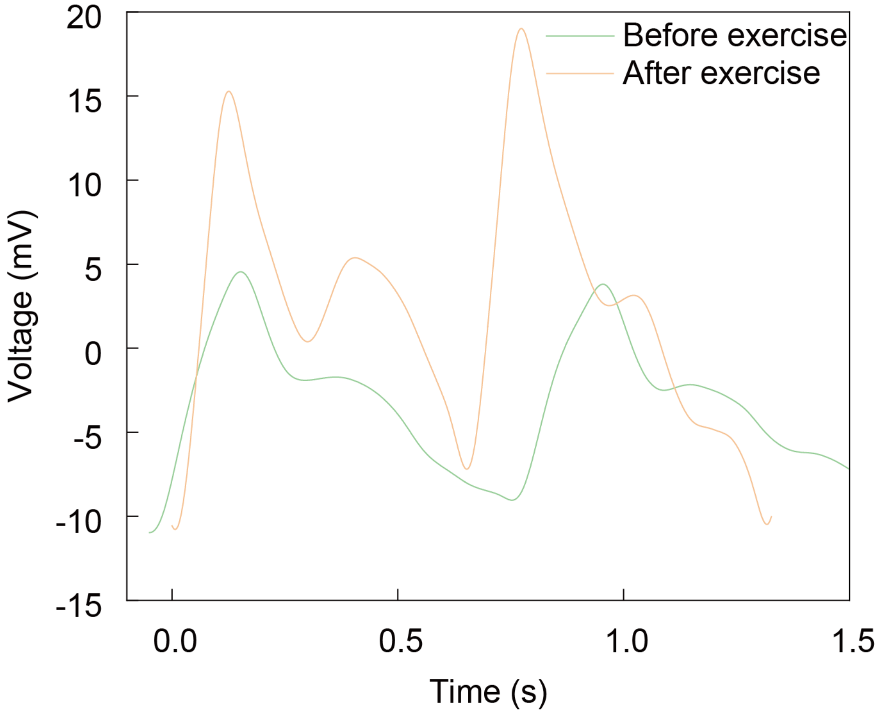

Supplementary Fig. 18. Wave form comparison before and after exercise.

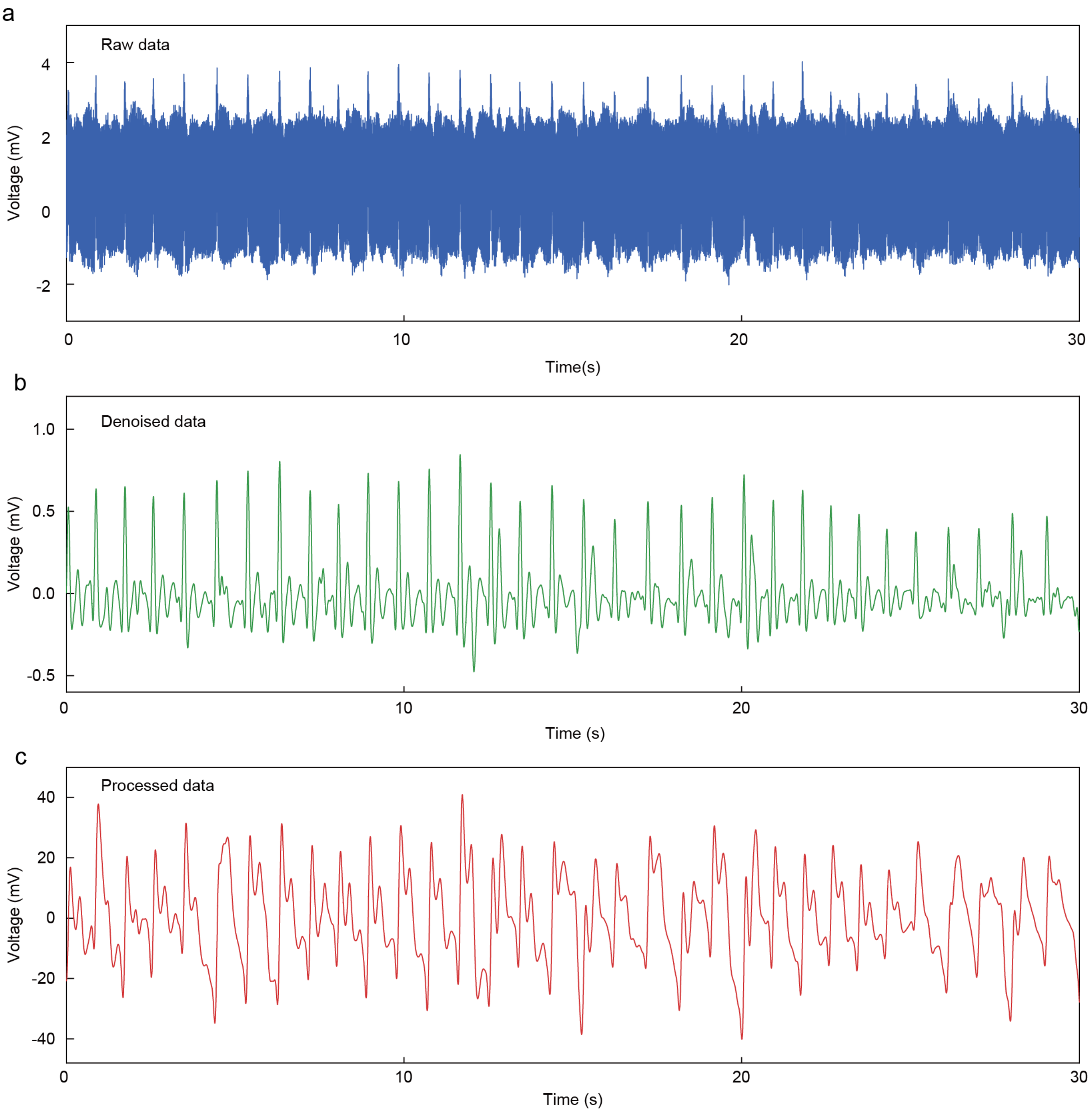

Supplementary Fig. 19. Signal preprocessing process. (a). Measured piezo response. (b). Filtered piezo response. (c). Integrated pulse waveform.

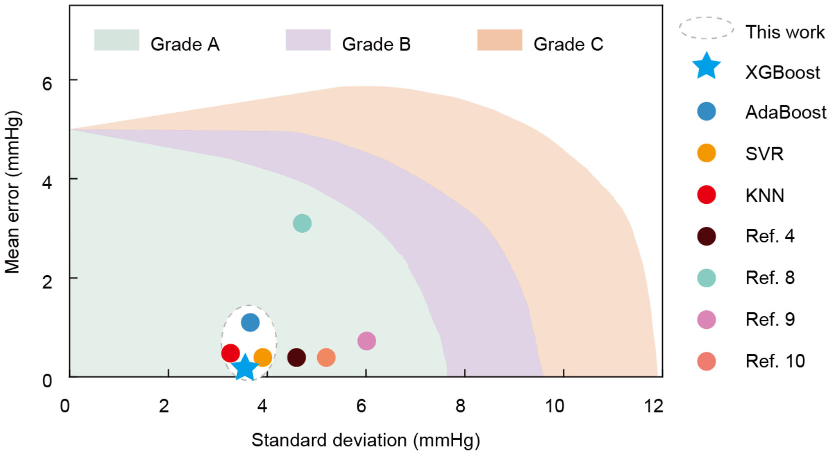

Supplementary Fig. 20. Performance comparison in DBP of different prediction algorithms utilized in this work (oval with white background) and other works, PPG[12], Bio-Z[4,13], Resistive pressure sensor[14], under the IEEE srandard for wearable BP monitoring devices.

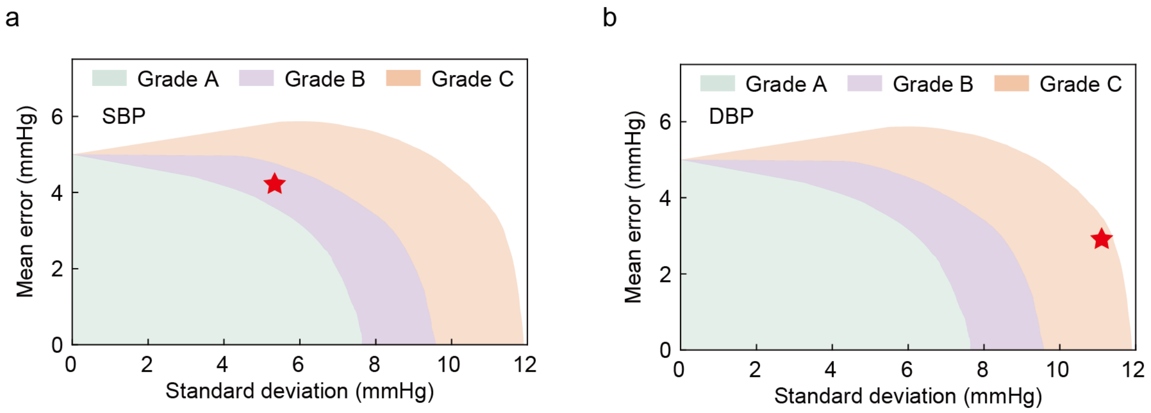

Supplementary Fig. 21. SBP (a) and DBP (b) estimation accuracy without local PWV involved in model training.

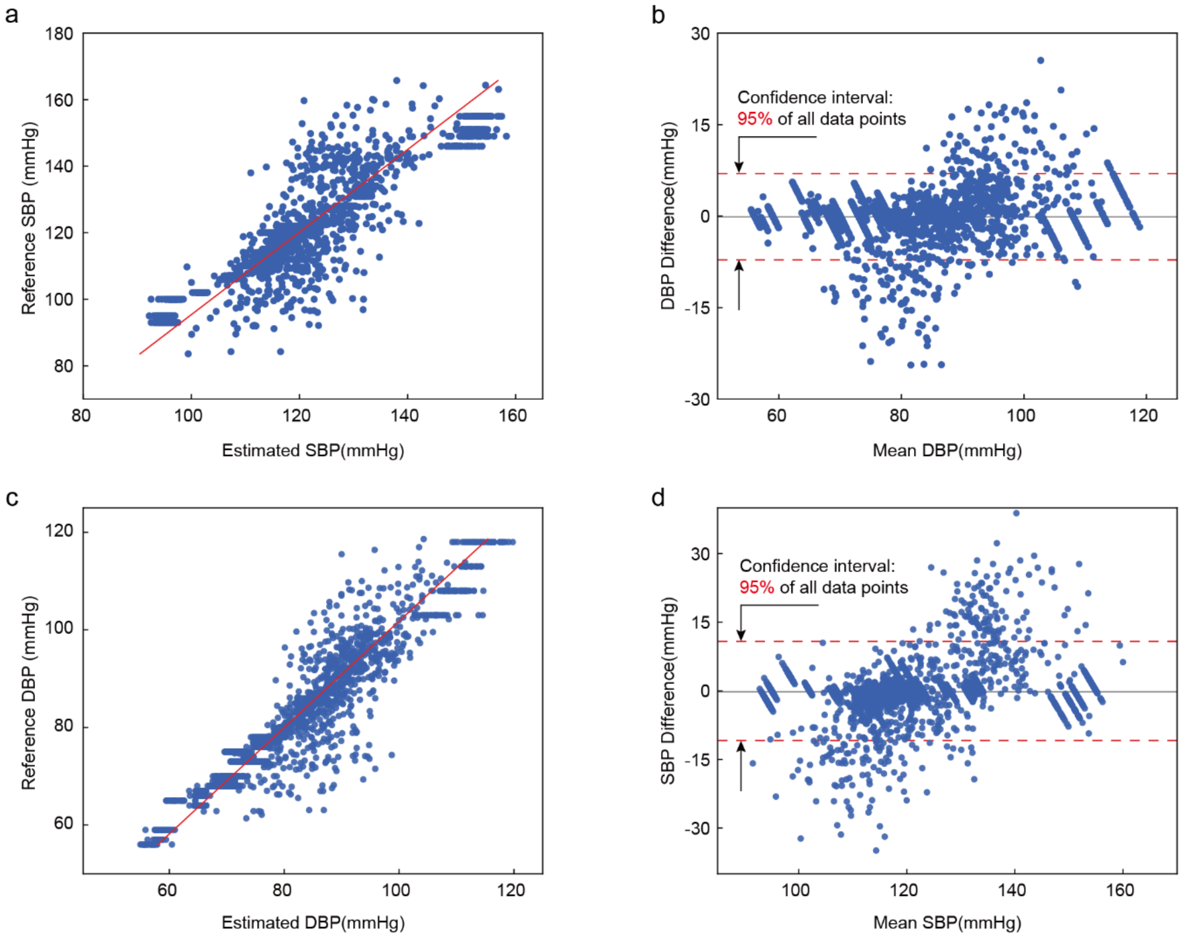

Supplementary Fig. 22. The correlation diagram and the Bland-Altman plots of blood pressure. (a) and (b) The correlation scatter plots between reference blood pressure and estimated values for SBP (a) and DBP (b). The diagonal is the linear regression line. r is the Pearsons coefficient. (c) and (d) The Bland–Altman plots of mean values for SBP (c) and DBP (c). The diagram shows 95% confidence limits of the difference between measurements and estimated blood pressure values.

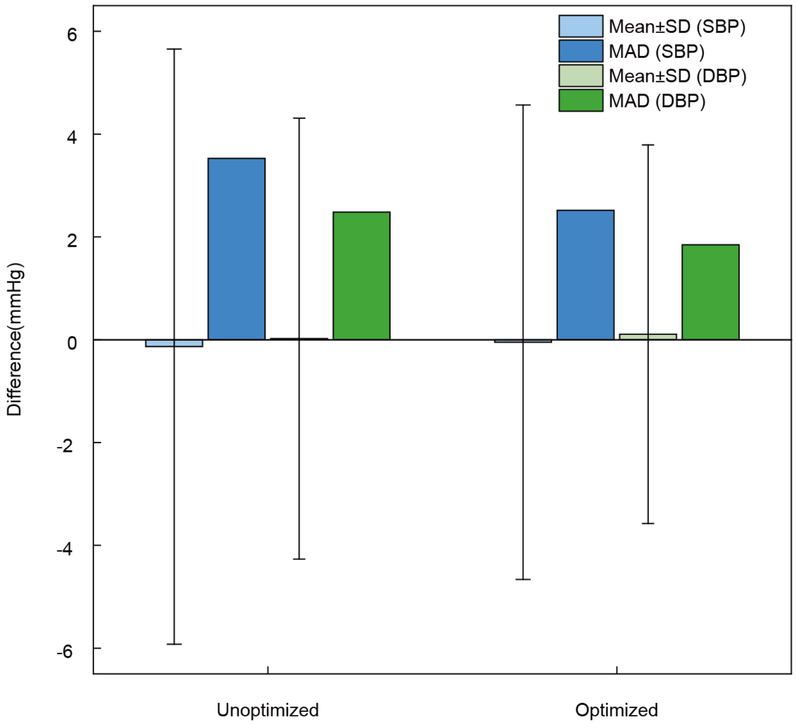

Supplementary Fig. 23. Performance comparison of the data before and after optimization.

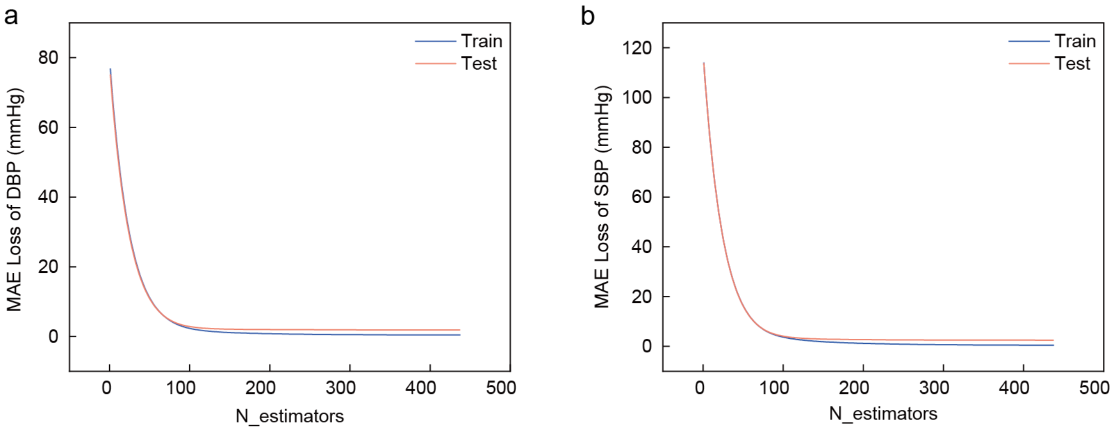

Supplementary Fig. 24. Loss curves during model training and validation process. (a). Loss curves of DBP prediction as estimators increases. (b). Loss curves of SBP prediction as the estimators increases.

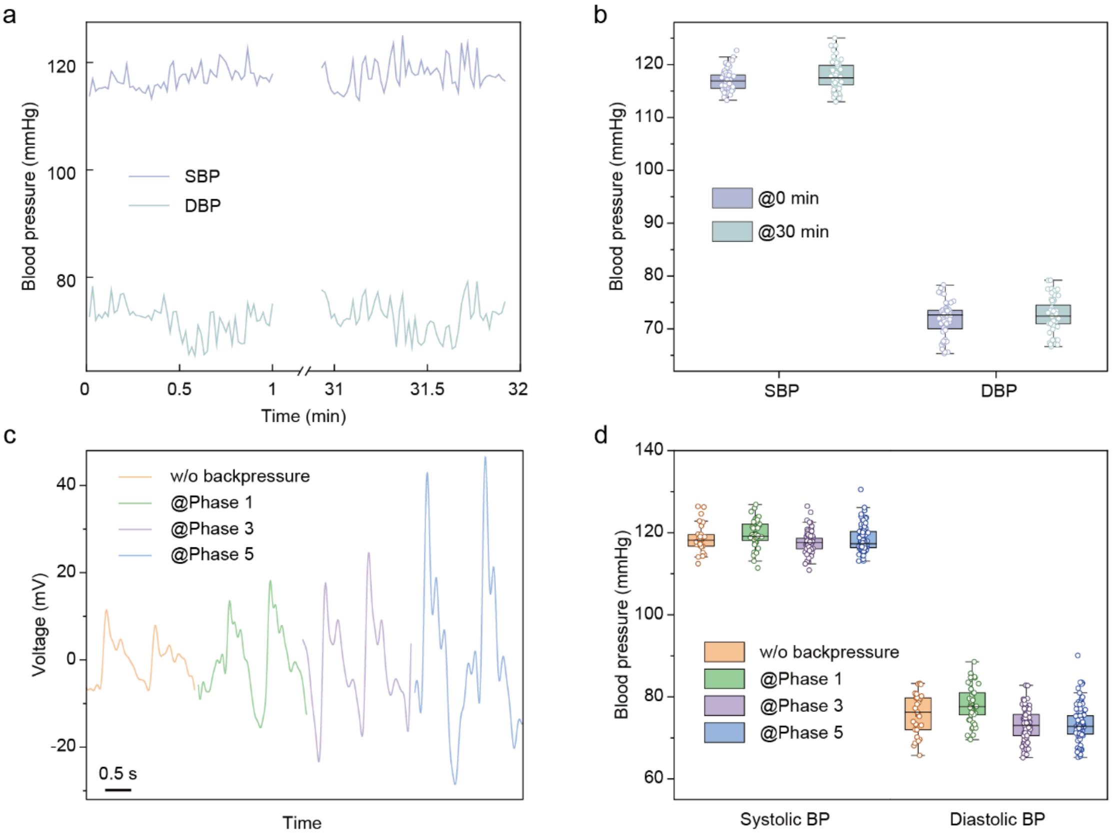

Supplementary Fig. 25. BP estimation performance validation and stability improvement with the active pressure adaption module. Continuous BP wave forms (a) and statistical BP (b) comparison for 1 minute between the micro airbag just pumped and 30 minutes after the inflation. Comparison on measured continuous pulse waves (c) and statistical BP (d) under different airbag pressure. n = 60 BP points; center line, median; box limits, upper and lower quartiles; whiskers, 1.5×interquartile range; points, overlapped data points in box plots of b and d.

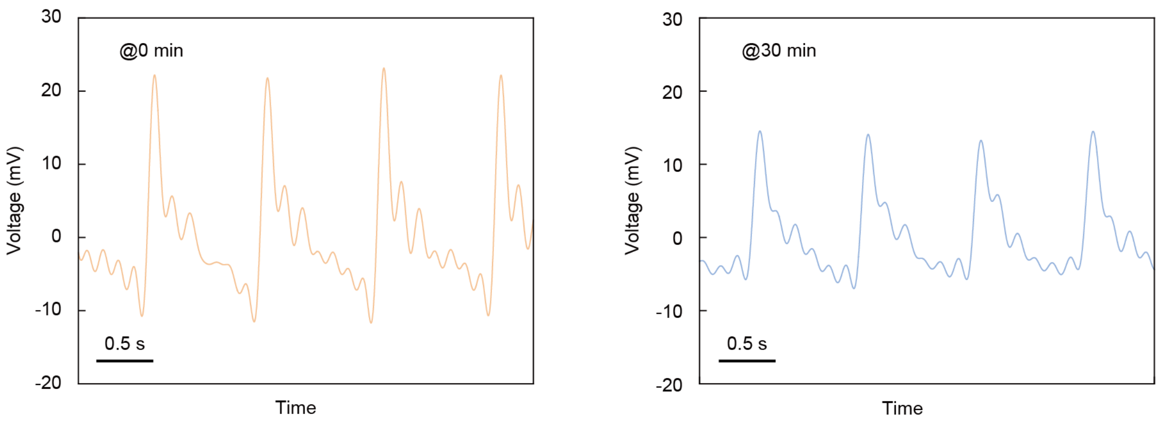

Supplementary Fig. 26. Comparison in measured pulse wave at 0 and 30 minutes after the inflation.

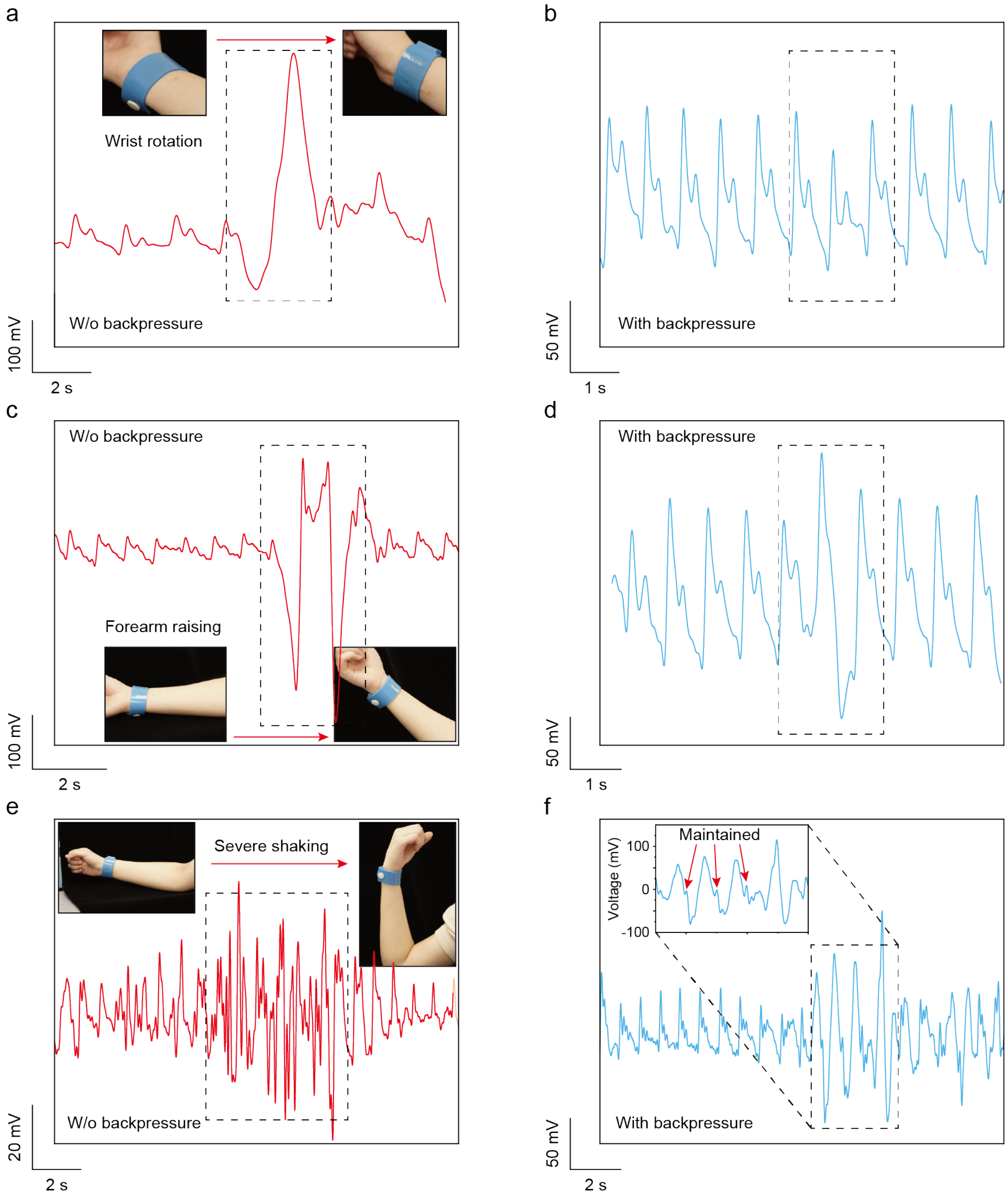

Supplementary Fig. 27. Performance validation of the active pressure adaptation module on interfacial stability enhancement. (a) and (b). Comparison on measured pulse signal with and without backpressure applied under 90° wrist rotation. (c) and (d). Comparison on measured pulse signal with and without backpressure applied under forearm raising. (e) and (f). Comparison on measured pulse signal with and without backpressure applied under severe forearm shaking.

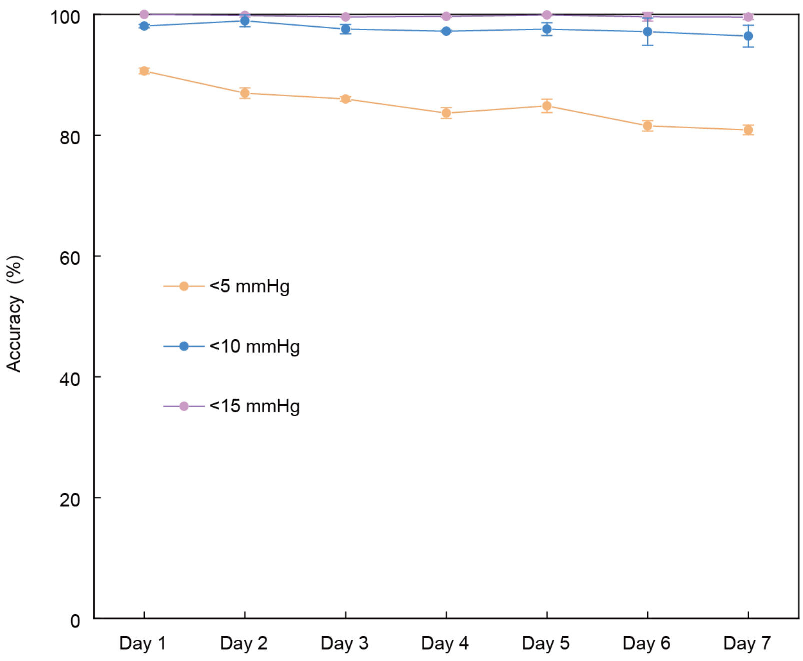

Supplementary Fig. 28. DBP accuracy evaluation in a week after calibration (n = 3 tests on the same volunteer; error bars, S.D.).

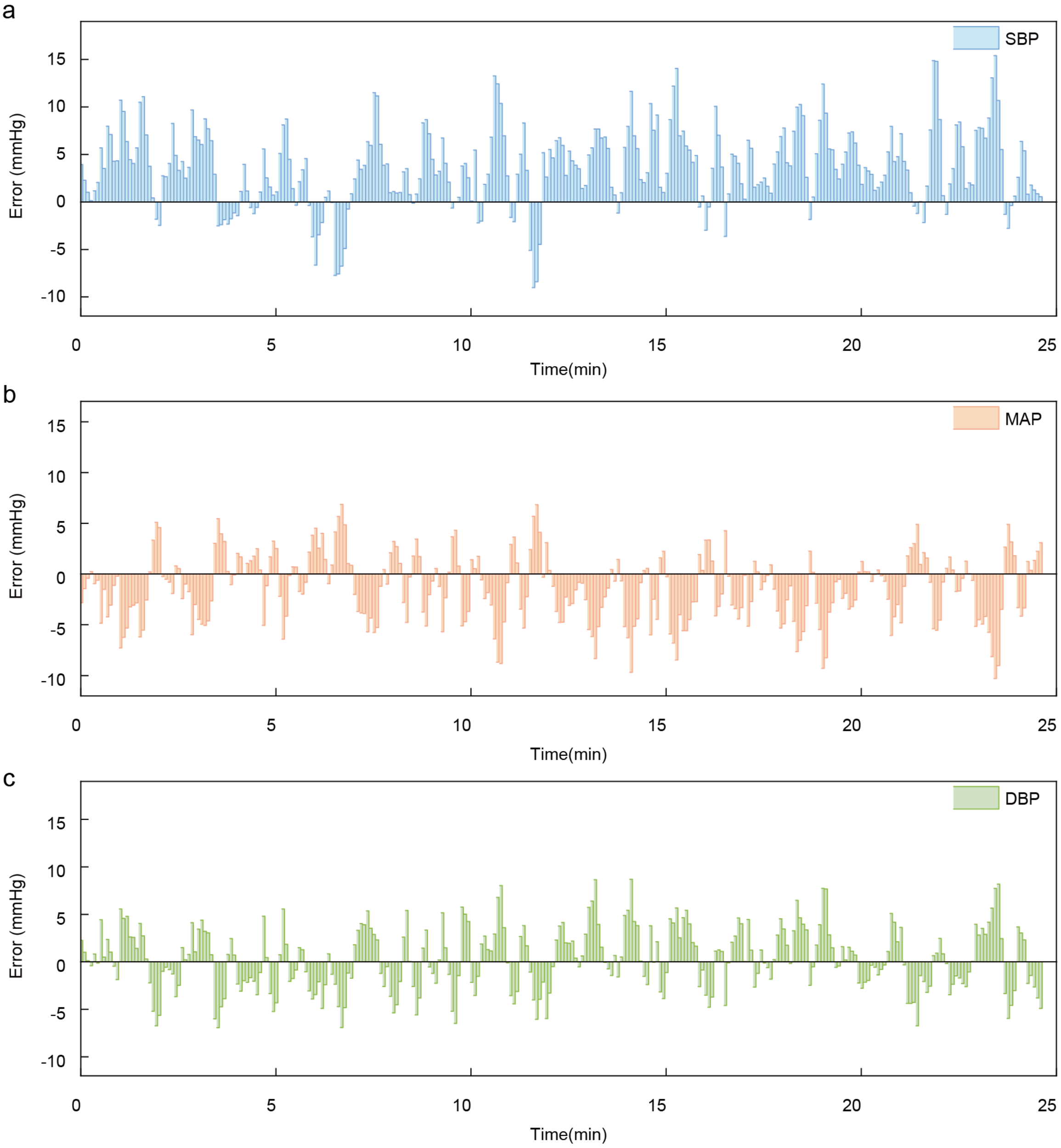

Supplementary Fig. 29. Error comparison of the TSMS in comparison with commercial BP monitoring system in daily office scene. Relative errors in SBP (a), MAP (b) and DBP (c) of the wireless system, where the maximum error in SBP and DBP are less than 15 mmHg and 10 mmHg, respectively, indicating the excellent performance.

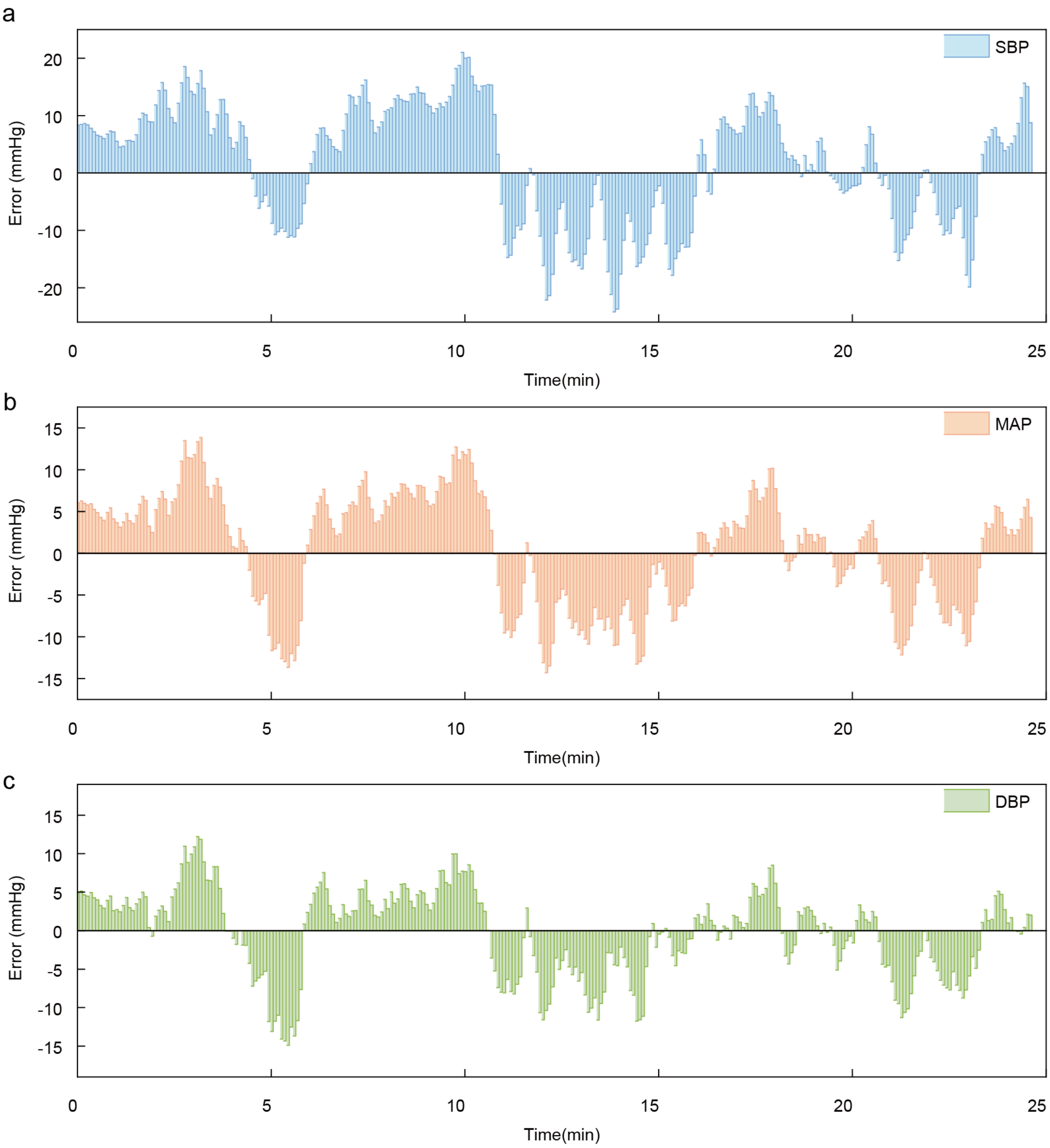

Supplementary Fig. 30. Error comparison of the wireless system in comparison with commercial Bio-PAC in HGCP maneuvers. Relative errors in SBP (a), MAP (b) and DBP (c) of the wireless system, where the maximum error in SBP and DBP are less than 20 mmHg and 15 mmHg, respectively, indicating the excellent performance.

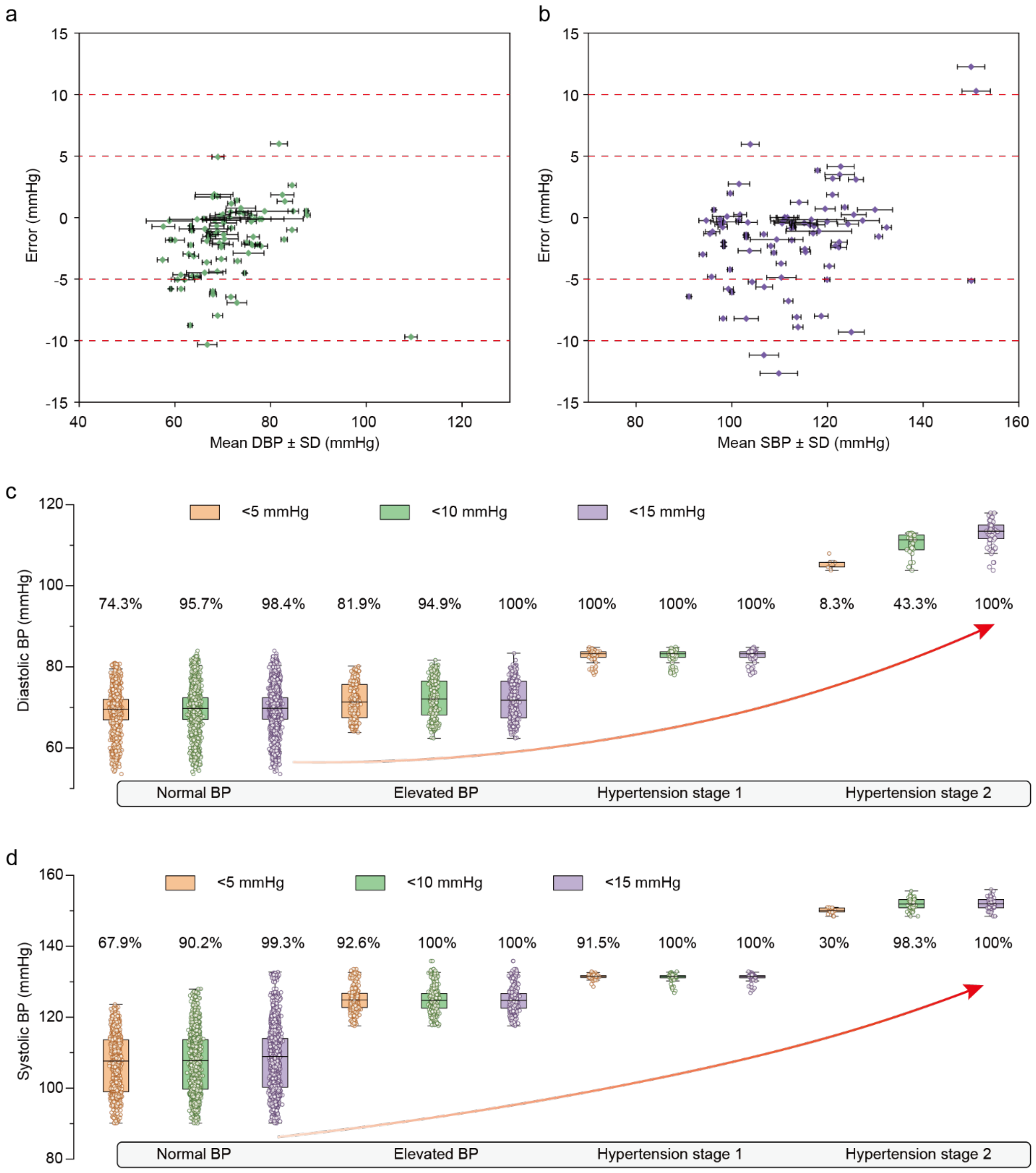

Supplementary Fig. 31. Quantitative statistics presenting the measurement accuracy of the TSMS on a total number of 87 volunteers. (a) and (b). Measurement accuracy comparison in DBP (a) and SBP (b) with commercial sphygmomanometer as a reference. Measurement accuracy statistics on systolic BP (c) and diastolic BP (d) representing the measurement accuracy in different BP categories under the standard of America Hypertension Association (AHA) with continuous BP measured by CNAP as reference. Square, mean; center line, median; box limits, upper and lower quartiles; whiskers, 1.5×interquartile range; points, data points, in box plots of c and d.

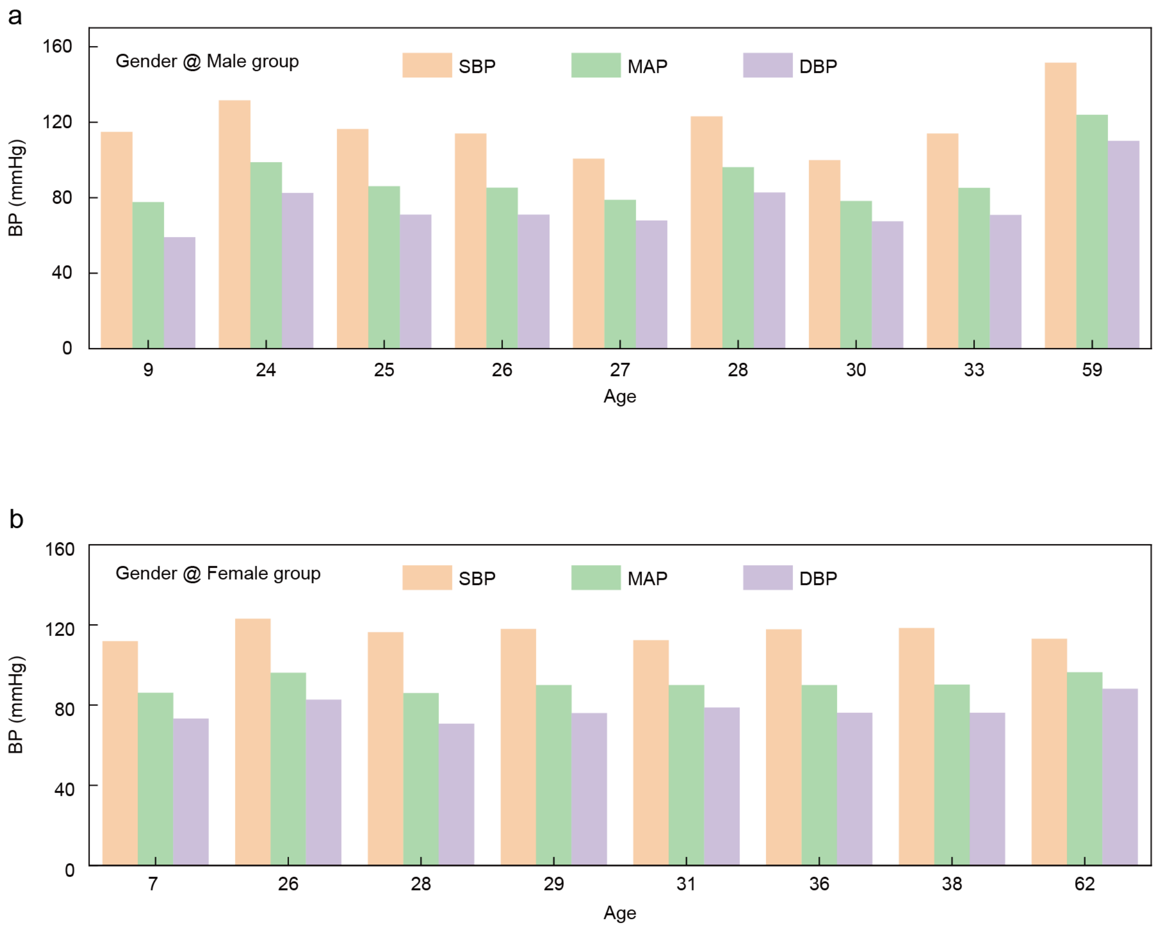

Supplementary Fig. 32. Statistical comparison of BP variation with increased age in male group and female group. (a). BP variation in male group, where a slight increase trend can be found with increased age. (b). BP variation in female group.

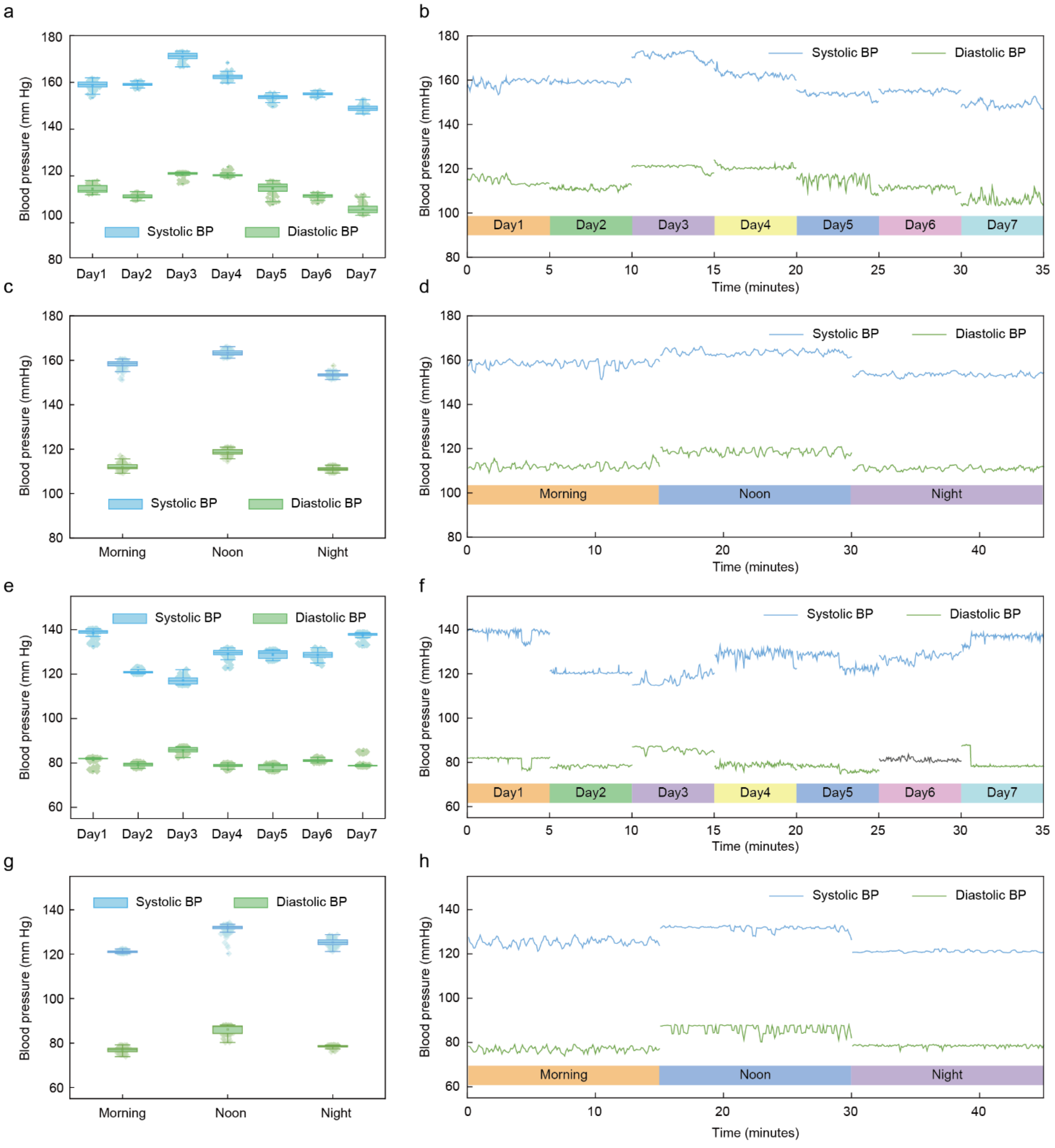

Supplementary Fig. 33. Continuous tracking and monitoring of two hypertension individuals for a week. (a) and (b). Statistical plots (a) and continuous waveform (b) present the change of SBP and DBP in a week of the hypertension individual, where 5 minutes continuous BP waveforms per day are presented. (c) and (d). Statistical plots (c) and continuous waveform (d) present the change of SBP and DBP in the morning, midday, and evening during a day of the hypertension individual, where 15 minutes continuous BP waveforms are presented for each slot. (e) and (f). Statistical plots (a) and continuous waveform (b) present the change of SBP and DBP in a week of the borderline hypertension individual, where 5 minutes continuous BP waveforms per day are presented. (g) and (h). Statistical plots (g) and continuous waveform (h) present the change of SBP and DBP in the morning, midday, and evening during a day of the borderline hypertension individual, where 15

minutes continuous BP waveforms are presented for each slot. $n = 77$ BP data points; center line, median; box limits, upper and lower quartiles; whiskers, $1.5\times$interquartile range; points, overlapped data points in box plots of a, c, e, and g.

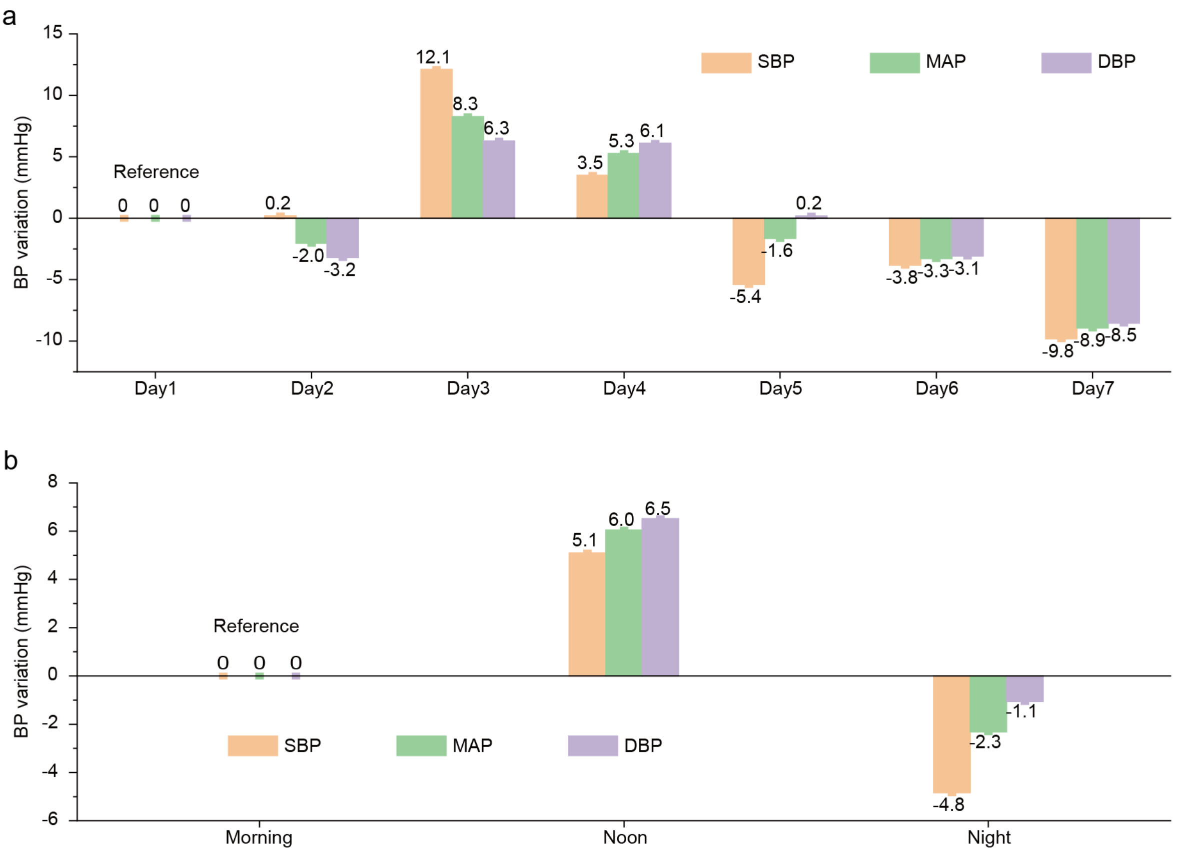

Supplementary Fig. 34. Statistical plot of BP variation of the hypertension individual. (a). BP variation during a week (a) and a day (b). The BP in day one and the morning are regarded as the reference.

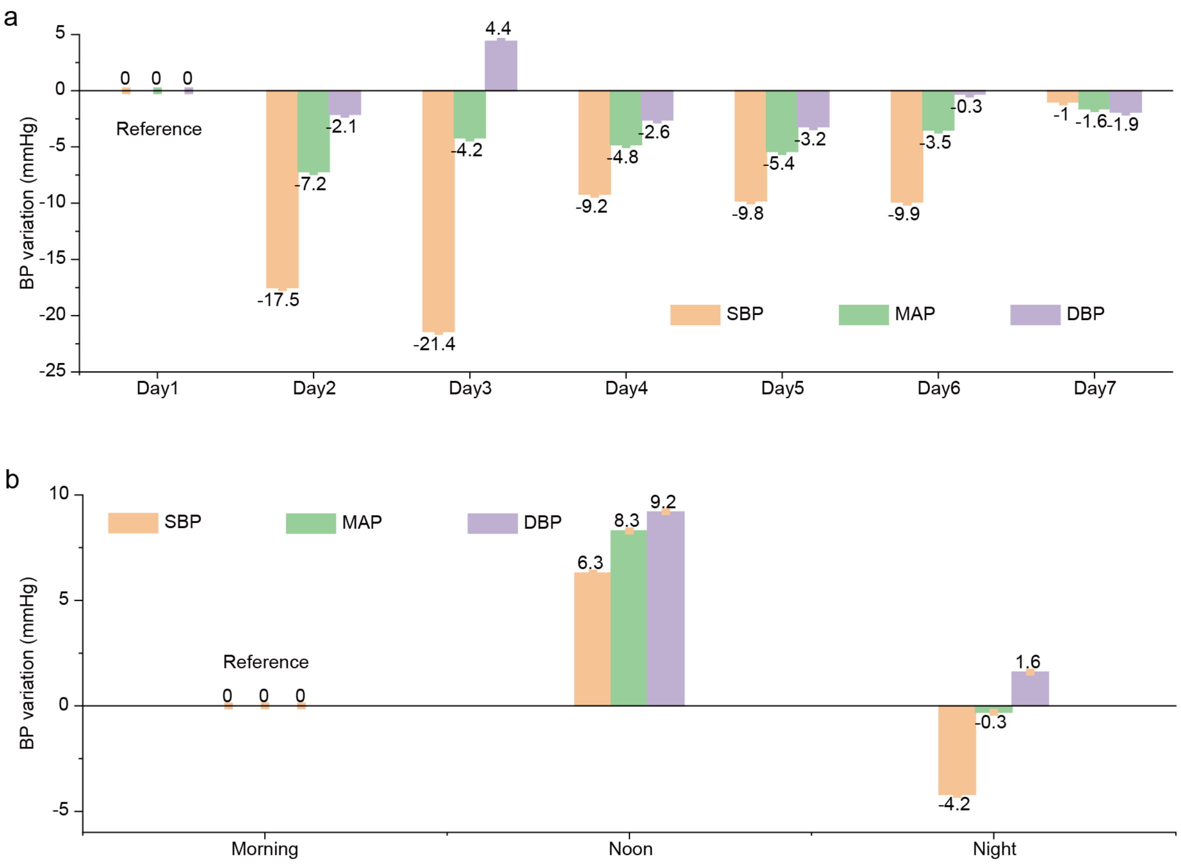

Supplementary Fig. 35. Statistical plot of BP variation of the borderline hypertension individual. (a). BP variation during a week (a) and a day (b). The BP in day one and the morning are regarded as the reference.

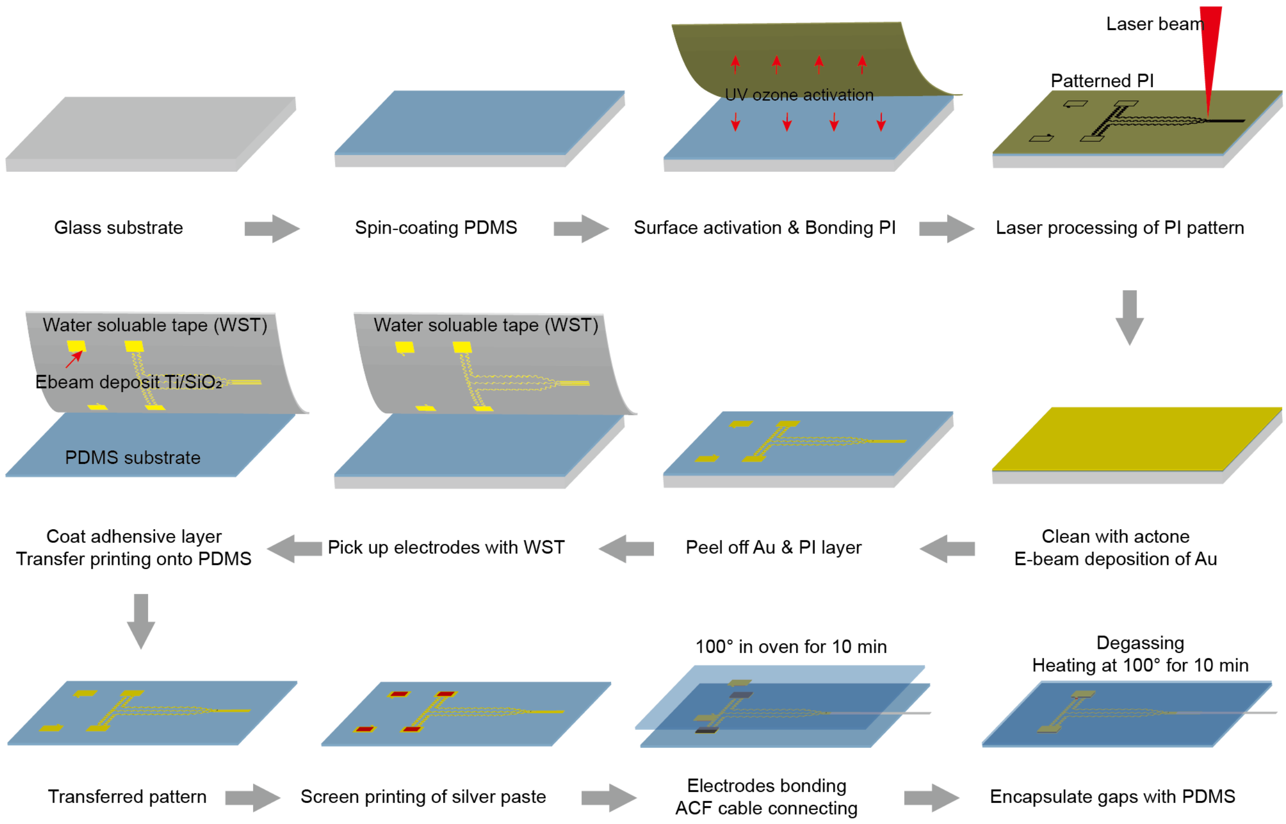

Supplementary Fig. 36. Fabrication process of the flexible sensor array.

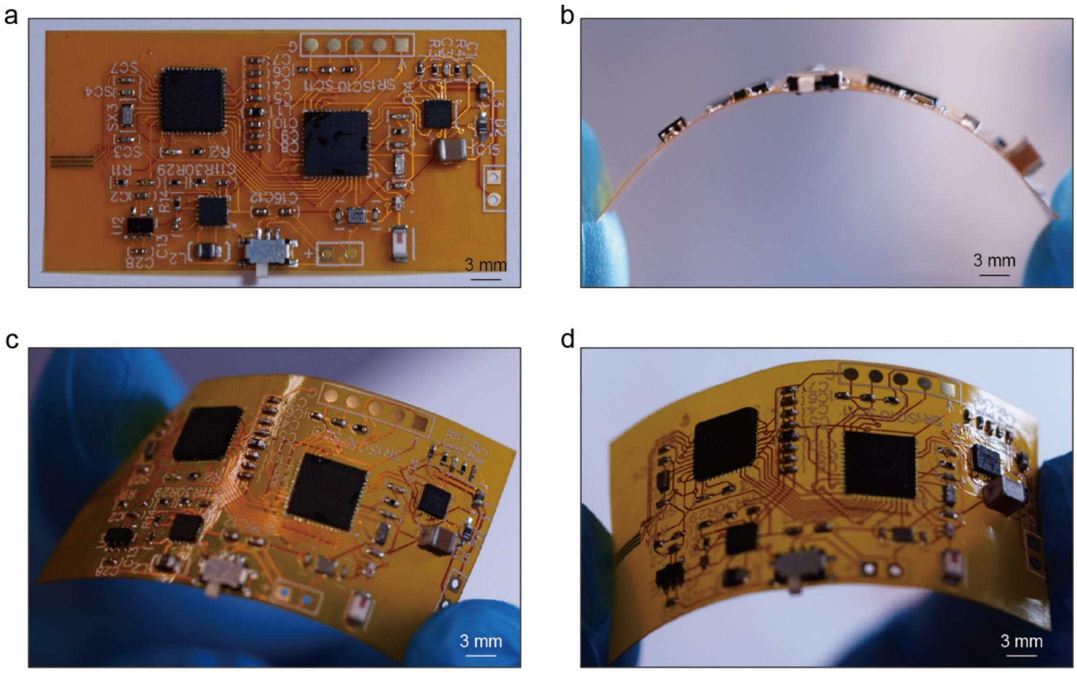

Supplementary Fig. 37. Optical images of the fPCB. (a) Top view of the fPCB and optical images of the fPCB under different deformation, bending (b, d) and twisting (c) showing its excellent flexibility.

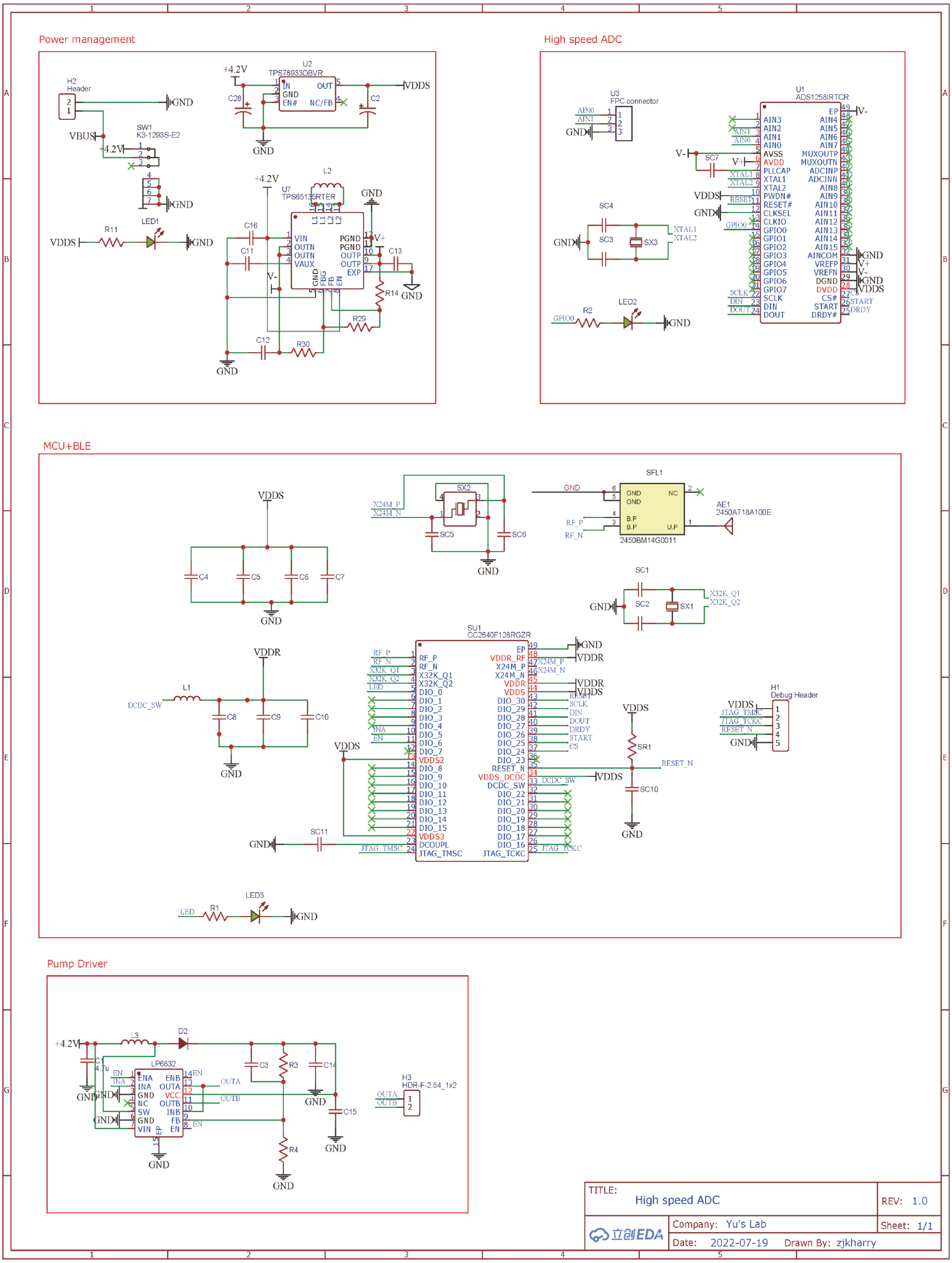

Supplementary Fig. 38. Schematic design of the signal sampling/processing module.

Supplementary Table 1. Technical comparison of continuous BP monitoring devices.

Method	Wireless? Totally wearable?	Continuous?	Accuracy DBP (mmHg) ME	SD	SBP (mmHg) ME	SD	Device dimension
PPG[15]	Yes<br>PPG sensor and hard sampling board in a watch shaped wrist.	Not	-0.07±4.47		0.00±3.61		Not reported.
PPG[12]	No<br>Commercial PPG sensor and ECG electrode required.	Yes<br>Beat to beat.<br>Static BP.	3.23±4.75		4.43±6.09		Not reported.
PPG[16]	Yes	Yes<br>1000s long.<br>Continuous.<br>Static BP.	Not reported.		0.24	1.18	Device size<br>40 mm × 20 mm<br>Board size<br>Not reported.
PPG[17]	Yes<br>Neither the sensor nor the electrical circuit are designed in wearable format.	Not	2.12	0.26	2.94	0.72	Not reported.
Piezoelectric ultrasound transducer[18]	No<br>Connecting to sampling equipment required.	Yes<br>30s long.<br>Continuous.<br>Static BP.	Not reported.				23 mm × 20 mm
Piezoelectric ultrasound transducer[1]	Yes<br>Yes	Yes<br>Static BP	0.17 ± 4.92				Device size not reported.<br>20.29 cm^3 battery for 12 h lifetime.
Resistive pressure sensor combined with ECG[14]	Yes<br>Wearable pulse sensor and ECG electrodes are worn separately.	Yes<br>4 hour long.<br>15s time window.<br>Static BP.	0.24	5.19	0.07	9.66	ECG patch<br>70 mm × 20 mm<br>Pulse sensor<br>40 mm × 17 mm
Piezoelectric pressure sensor[6]	No<br>Sensor in wearable format.<br>Rigid sampling board.<br>Sensor and electrical board were not integrated.	Yes<br>Static BP.	Not reported.		Not reported.		Device size 3.5 mm in diameter.<br>Board size not reported.
Piezoelectric pressure sensor[5]	Yes<br>Yes	Yes<br>Static BP.	-0.89	6.19	-0.32	5.28	Device size not reported.<br>Integrated in a wristwatch.<br>Watch size not reported.
Capacitive pressure sensor[19]	No<br>Connecting to sampling equipment	Yes<br>10 min. long.<br>Static BP	0.48	1.96	1.43	1.96	8 mm × 8 mm

	required.						
Capacitive pressure sensor[20]	No<br>Fixing wristband and sampling equipment required.	Yes<br>9 min. long. 70 beats window.<br>Static BP	-0.054 ± 2.09				15 mm × 2 mm
Commercial silver electrodes. Bio-impedance[13]	No<br>Bulky wristband and sampling board required.	Yes<br>60 min. long. 5-10s time window.<br>Dynamic BP	-1.3	6	3.7	8.5	Device size 64 mm × 46 mm<br>Board size not reported.
Graphene tattoo[4] Bio-impedance	No<br>Extra connecting wires required from the graphene tattoo to the sampling board.	Yes<br>5+ hours long. 5-10s time window.<br>Dynamic BP	0.2	4.5	0.2	5.8	200 nm in thickness. Extra sampling board required.
**Piezoelectric pressure sensor ★**	Yes<br>Soft sensor together with flexible sampling board encapsulated in a thin wristband.	Yes<br>All day.<br>Beat by beat.<br>Dynamic BP	0.11	3.68	-0.05	4.61	Planar size: 150 mm × 350 mm<br>Thickness 4 mm

★ This work.

Dynamic BP and static BP are defined as whether BP fluctuations were created during the measurement.

Supplementary Table 2. Definition of input features.

No.	Features	Description
1	T	Time of one pulse waveform
2	T1	Systolic peak time
3	T2	Dicrotic notch time
4	T3	Diastolic peak time
5	Tpp	Pulse peak-to-peak interval
6	PTT	Pulse transit time between two pulse waves
7~12	ST10, ST25, ST33, ST50, ST66, ST75	10%, 25%, 33%, 50%, 66%, 75% of systolic time span
13~18	DT10, DT25, DT33, DT50, DT66, DT75	10%, 25%, 33%, 50%, 66%, 75% of diastolic time span
19	x	Systolic peak height
20	y	Diastolic peak height
21	(x-y)/x	Relative augmentation index
22	A2/A1	Inflection point area ratio; A1: the areas under the curve between the systolic foot and systolic peak; A2: the area under the curve between the systolic peak and dicrotic notch;
23	HR	Mean heart rate during the experiment
24	Age	Age of the participant
25	Gender	Gender of the participant (0 for male and 1 for female)
26	BMI	Body mass index: weight(kg) divided by the square of height (m)

Supplementary Table 3: Volunteer information

No.	Age	Gender (Male/Female)	Height (cm)	Weight (kg)	BMI (Kg/m^2)	Average HR (Beats/min)
1	7	Female	124	28	18.2	87
2	9	Male	149	41	18.5	84
3	24	Male	184	90	26.6	72
4	25	Male	178	68	21.5	68
5	26	Male	175	70	22.9	62
6	26	Female	163	51	19.2	83
7	27	Male	187	91	26	71
8	28	Female	160	50	19.5	84
9	28	Male	172	61	21	61
10	29	Female	160	51	19.9	90
11	30	Male	172	73	24.7	77
12	31	Female	167	60	21.5	93
13	33	Male	170	59	20.4	84
14	36	Female	165	64	23.5	80
15	38	Female	160	61	23.8	73
16	59	Male	185	71	20.7	57
17	62	Female	155	58	24.1	74

Supplementary Table 4. Model optimization parameters

Hyperparameters	Search range	Optimized results
Colsample_bytree	(0.4, 1)	0.9397
Gamma	(0.1, 10)	3.2650
Max_depth	(6, 15)	13
N_estimators	(150, 450)	437
Learning_rate	(0.01, 0.8)	0.0393
Min_child_weight	(1, 20)	2.4860
Reg_alpha	(0, 1)	0.0063
Subsample	(0.5, 1)	0.7787

Supplementary References

1. Lin, M. *et al.* A fully integrated wearable ultrasound system to monitor deep tissues in moving subjects. *Nat Biotechnol* 1–10 (2023) doi:10.1038/s41587-023-01800-0.

2. Palombo, C. & Kozakova, M. Arterial stiffness, atherosclerosis and cardiovascular risk: Pathophysiologic mechanisms and emerging clinical indications. *Vascular Pharmacology* **77**, 1–7 (2016).

3. Wada, T., Fujishiro, K., Fukumoto, T., Yamazaki, S. & Wada, T. Relationship Between Ultrasound Assessment of Arterial Wall Properties and Blood Pressure. *Angiology* **48**, 893–900 (1997).

4. Kireev, D. *et al.* Continuous cuffless monitoring of arterial blood pressure via graphene bioimpedance tattoos. *Nat. Nanotechnol.* 1–7 (2022) doi:10.1038/s41565-022-01145-w.

5. Min, S. *et al.* Clinical Validation of a Wearable Piezoelectric Blood-Pressure Sensor for Continuous Health Monitoring. *Advanced Materials* **n/a**, 2301627.

6. Yi, Z. *et al.* Piezoelectric Dynamics of Arterial Pulse for Wearable Continuous Blood Pressure Monitoring. *Advanced Materials* **n/a**, 2110291.

7. Yang, J. *An Introduction to the Theory of Piezoelectricity*. (Springer, 2005).

8. Arakawa, M., Kudo, K., Kobayashi, K. & Kanai, H. Blood pressure measurement using piezoelectric effect by an ultrasonic probe. *Sensors and Actuators A: Physical* **286**, 146–151 (2019).

9. Goldstein, D. S. & Cheshire, W. P. Beat-to-beat blood pressure and heart rate responses to the Valsalva maneuver. *Clin Auton Res* **27**, 361–367 (2017).

10. Prakash, E. S., Madanmohan, null, Sethuraman, K. R. & Narayan, S. K. Cardiovascular autonomic regulation in subjects with normal blood pressure, high-normal blood pressure and recent-onset hypertension. *Clin Exp Pharmacol Physiol* **32**, 488–494 (2005).

11. Lamotte, G., Boes, C. J., Low, P. A. & Coon, E. A. The expanding role of the cold pressor test: a brief history. *Clin Auton Res* **31**, 153–155 (2021).

12. Yang, S., Zhang, Y., Cho, S.-Y., Correia, R. & Morgan, S. P. Non-invasive cuff-less blood pressure estimation using a hybrid deep learning model. *Opt Quant Electron* **53**, 93 (2021).

13. Ibrahim, B. & Jafari, R. Cuffless blood pressure monitoring from a wristband with calibration-free algorithms for sensing location based on bio-impedance sensor array and autoencoder. *Sci Rep* **12**, 319 (2022).

14. Gong, S. *et al.* A gold nanowire-integrated soft wearable system for dynamic continuous non-invasive cardiac monitoring. *Biosensors and Bioelectronics* **205**, 114072 (2022).

15. Elgendi, M. *et al.* The use of photoplethysmography for assessing hypertension. *npj Digit. Med.* **2**, 1–11 (2019).

16. Li, H. *et al.* Wearable skin-like optoelectronic systems with suppression of motion artifacts for cuff-less continuous blood pressure monitor. *National Science Review* **7**, 849–862 (2020).

17. Byfield, R., Miller, M., Miles, J., Guidoboni, G. & Lin, J. Towards Robust Blood Pressure Estimation From Pulse Wave Velocity Measured by Photoplethysmography Sensors. *IEEE Sensors Journal* **22**, 2475–2483 (2022).

18. Wang, C. *et al.* Monitoring of the central blood pressure waveform via a conformal ultrasonic device. *Nat Biomed Eng* **2**, 687–695 (2018).

19. Rwei, P. *et al.* Soft Iontronic Capacitive Sensor for Beat-to-Beat Blood Pressure Measurements. *Advanced Materials Interfaces* **9**, 2200294 (2022).

20. Kim, J. *et al.* Soft Wearable Pressure Sensors for Beat-to-Beat Blood Pressure Monitoring. *Advanced Healthcare Materials* **8**, 1900109 (2019).
