# Supplementary material for: Thin, soft, wearable system for continuous wireless monitoring of artery blood pressure
Source: Nat Commun. 2023 Aug 17;14:5009. doi: 10.1038/s41467-023-40763-3 (PMC10435523; doi:10.1038/s41467-023-40763-3)
Supplement: Supplementary file 3 — Description of additional supplementary files [file 41467_2023_40763_MOESM3_ESM.pdf]

Description of Additional Supplementary Files Document

Supplementary Movie 1: Real-time shape changes of the micro airbag driven by the micro pump with the pumping pressure of 10 KPa.

Supplementary Movie 2: Performance of comparison of the wireless wristband by commercial continuous BP monitoring equipment (Bio-PAC). The inset video shows the measured continuous BP pattern during the process.
