# Supplementary material for: Thin, soft, wearable system for continuous wireless monitoring of artery blood pressure
Source: Nat Commun. 2023 Aug 17;14:5009. doi: 10.1038/s41467-023-40763-3 (PMC10435523; doi:10.1038/s41467-023-40763-3)
Supplement: Supplementary file 6 — Reporting Summary [file 41467_2023_40763_MOESM6_ESM.pdf]

nature portfolio

Corresponding author(s): Xiaohui Ma and Xinge Yu

Last updated by author(s): Jul 29, 2023

Reporting Summary

Nature Portfolio wishes to improve the reproducibility of the work that we publish. This form provides structure for consistency and transparency in reporting. For further information on Nature Portfolio policies, see our Editorial Policies and the Editorial Policy Checklist.

Statistics

For all statistical analyses, confirm that the following items are present in the figure legend, table legend, main text, or Methods section.

n/a	Confirmed	
☐	☒	The exact sample size (n) for each experimental group/condition, given as a discrete number and unit of measurement
☐	☒	A statement on whether measurements were taken from distinct samples or whether the same sample was measured repeatedly
☒	☐	The statistical test(s) used AND whether they are one- or two-sided<br>*Only common tests should be described solely by name; describe more complex techniques in the Methods section.*
☒	☐	A description of all covariates tested
☒	☐	A description of any assumptions or corrections, such as tests of normality and adjustment for multiple comparisons
☐	☒	A full description of the statistical parameters including central tendency (e.g. means) or other basic estimates (e.g. regression coefficient) AND variation (e.g. standard deviation) or associated estimates of uncertainty (e.g. confidence intervals)
☒	☐	For null hypothesis testing, the test statistic (e.g. F, t, r) with confidence intervals, effect sizes, degrees of freedom and P value noted<br>*Give P values as exact values whenever suitable.*
☒	☐	For Bayesian analysis, information on the choice of priors and Markov chain Monte Carlo settings
☒	☐	For hierarchical and complex designs, identification of the appropriate level for tests and full reporting of outcomes
☐	☒	Estimates of effect sizes (e.g. Cohen's d, Pearson's r), indicating how they were calculated

Our web collection on statistics for biologists contains articles on many of the points above.

Software and code

Policy information about availability of computer code

Data collection	Powerlab by AD instrument, DAQ 6510 data acquisition system, Self-developed control panel for voltage measurement, CNAP monitor 500 connected to BioPAC (systems, Inc) for continuous blood pressure measurement, Omron HEM-7156T for isolated blood pressure measurement. Abaqus (2018) was utilized for piezoelectric material simulation.
Data analysis	Matlab R2018a, Self-developed data model for blood pressure estimation.

For manuscripts utilizing custom algorithms or software that are central to the research but not yet described in published literature, software must be made available to editors and reviewers. We strongly encourage code deposition in a community repository (e.g. GitHub). See the Nature Portfolio guidelines for submitting code & software for further information.

Data

Policy information about availability of data

All manuscripts must include a data availability statement. This statement should provide the following information, where applicable:

- Accession codes, unique identifiers, or web links for publicly available datasets
- A description of any restrictions on data availability
- For clinical datasets or third party data, please ensure that the statement adheres to our policy

The data that support the findings of this study are available from the corresponding authors upon request.

Research involving human participants, their data, or biological material

Policy information about studies with human participants or human data. See also policy information about sex, gender (identity/presentation), and sexual orientation and race, ethnicity and racism.

Reporting on sex and gender	In this study, we recruited 87 volunteers (39 male and 48 female) for volunteer experiments. Gender was not a factor considered in the experiment design. All the volunteers were recruited for continuous and isolated blood pressure measurement for evaluation of the device performance, where gender is not a variable that affects the results.
Reporting on race, ethnicity, or other socially relevant groupings	All the human subjects were recruited randomly and were not categorized by race, ethnicity, national or any other socially constructed or socially relevant groupings.
Population characteristics	87 volunteers with 39 male and 48 female in a reasonable range of age and BMI are recruited.
Recruitment	We posted flyers in the university and volunteers contacted us by themselves. Each volunteer was paid 100 HKD.
Ethics oversight	Human and Artefacts Ethics Sub-Committee, City University of Hong Kong with the reference number of HU-STA-00000393. The informed consent of all participants was obtained prior inclusion in this study..

Note that full information on the approval of the study protocol must also be provided in the manuscript.

Field-specific reporting

Please select the one below that is the best fit for your research. If you are not sure, read the appropriate sections before making your selection.

☒ Life sciences ☐ Behavioural & social sciences ☐ Ecological, evolutionary & environmental sciences

For a reference copy of the document with all sections, see nature.com/documents/nr-reporting-summary-flat.pdf

Life sciences study design

All studies must disclose on these points even when the disclosure is negative.

Sample size	87 volunteers with 39 male and 48 female in a reasonable range of age and BMI are recruited. A minimum sample size of 80 is required by British Hypertension Society protocol for the evaluation of blood pressure measuring devices
Data exclusions	No data was excluded from analysis.
Replication	All the measurements were taken for more than 3 times and all the attempts at replication were successful. All the testing results showed high similarity, and the reproducibility of all kinds of measurements were good in this work.
Randomization	The human subjects were recruited and selected randomly by posting flyers and they were randomly allocated into experiment group.
Blinding	All the human subjects were only told the experiment process. They did not involved in data process and none of them knew the testing results. Blinding to the investigators were not required since there was no subjective data related to volunteers need to be recorded.

Reporting for specific materials, systems and methods

We require information from authors about some types of materials, experimental systems and methods used in many studies. Here, indicate whether each material, system or method listed is relevant to your study. If you are not sure if a list item applies to your research, read the appropriate section before selecting a response.

Materials & experimental systems

n/a	Involved in the study	
☒	☐	Antibodies
☒	☐	Eukaryotic cell lines
☒	☐	Palaeontology and archaeology
☒	☐	Animals and other organisms
☒	☐	Clinical data
☒	☐	Dual use research of concern
☒	☐	Plants

Methods

n/a	Involved in the study	
☒	☐	ChIP-seq
☒	☐	Flow cytometry
☒	☐	MRI-based neuroimaging
